# A stochastic-field description of finite-size spiking neural networks

**DOI:** 10.1371/journal.pcbi.1005691

**Published:** 2017-08-07

**Authors:** Grégory Dumont, Alexandre Payeur, André Longtin

**Affiliations:** 1 Group for Neural Theory, Ecole Normale Supérieure, 29 rue d’Ulm, 75005, Paris, France; 2 Department of Physics, University of Ottawa, 150 Louis-Pasteur, Ottawa, Canada; 3 Centre for Neural Dynamics, Ottawa, Canada; University of Pittsburgh, UNITED STATES

## Abstract

Neural network dynamics are governed by the interaction of spiking neurons. Stochastic aspects of single-neuron dynamics propagate up to the network level and shape the dynamical and informational properties of the population. Mean-field models of population activity disregard the finite-size stochastic fluctuations of network dynamics and thus offer a deterministic description of the system. Here, we derive a stochastic partial differential equation (SPDE) describing the temporal evolution of the finite-size refractory density, which represents the proportion of neurons in a given refractory state at any given time. The population activity—the density of active neurons per unit time—is easily extracted from this refractory density. The SPDE includes finite-size effects through a two-dimensional Gaussian white noise that acts both in time and along the refractory dimension. For an infinite number of neurons the standard mean-field theory is recovered. A discretization of the SPDE along its characteristic curves allows direct simulations of the activity of large but finite spiking networks; this constitutes the main advantage of our approach. Linearizing the SPDE with respect to the deterministic asynchronous state allows the theoretical investigation of finite-size activity fluctuations. In particular, analytical expressions for the power spectrum and autocorrelation of activity fluctuations are obtained. Moreover, our approach can be adapted to incorporate multiple interacting populations and quasi-renewal single-neuron dynamics.

## Author summary

In the brain, information about stimuli is encoded in the timing of action potentials produced by neurons. An understanding of this neural code is facilitated by the use of a well-established method called mean-field theory. Over the last two decades or so, mean-field theory has brought an important added value to the study of emergent properties of neural circuits. Nonetheless, in the mean-field framework, the thermodynamic limit has to be taken, that is, to postulate the number of neurons to be infinite. Doing so, small fluctuations are neglected, and the randomness so present at the cellular level disappears from the description of the circuit dynamics. The origin and functional implications of variability at the network scale are ongoing questions of interest in neuroscience. It is therefore crucial to go beyond the mean-field approach and to propose a description that fully entails the stochastic aspects of network dynamics. In this manuscript, we address this issue by showing that the dynamics of finite-size networks can be represented by stochastic partial differential equations.

## Introduction

Neurons communicate by sending and receiving pulses called spikes which occur in a rather stochastic fashion. A stimulus is thus translated by neurons into spike trains with a certain randomness [1]. At the microscopic scale, this variability is mostly attributed to the probabilistic nature of the opening and closing of ion channels underlying the emission of an action potential. At a mesoscopic scale, variability typically stems from the seemingly random barrage of synaptic inputs. This variability is fundamentally noise [2, 3]. Many papers have been devoted to establish its origin [4, 5], and the mathematical formalization to suitably describe this effect has been an intense subject of research over the past decades. Nowadays, models are capable of reproducing both the statistics of the spiking activity and the subthreshold dynamics of different cell types.

In neuroscience, it is believed that information—about external stimuli, internal states, motor outputs, etc.—is encoded in the timing of spikes produced by populations of neurons [6]. An understanding of this high-dimensional response, from an information-theoretic or dynamical point of view, is facilitated by various dimensionality-reduction methods [7]. A trivial one is to consider the population activity, i.e. the proportion of neurons firing in small time windows, which is assumed to be a meaningful coarse-grained dynamical variable [6, 8–10]. The importance of population activity is manifest in its extensive use in macroscopic models of neural activity, and by the constant effort put forth to derive its dynamics from single-neuron and network properties.

Most attempts to produce analytically tractable population rate models have made use (directly or indirectly) of mean-field theory [11–14]. The population activity obtained by solving mean-field models is deterministic, since this theory neglects finite-size fluctuations. Theoretically, this causes no problem. Real neural circuits, however, necessarily have a finite size. For a system made up of *N* independent units, the relative magnitude of fluctuations should scale as $1/\sqrt{N}$. The thermodynamic limit (*N* goes to infinity) neglects those small fluctuations, and the randomness so present at the cellular level disappears in the description of the circuit. However, these finite-size effects should be taken into account because they can drastically affect both the synchronization [15, 16] and the stability [17, 18] of neural systems.

Various analytical methods from statistical physics have been used to describe such activity fluctuations. A first type of approach consists in adapting the Fokker-Planck formalism to incorporate the finite-size rate fluctuations as a source term [18, 19]. The spiking processes of the neurons are then assumed to be Poisson processes. One can also apply the so-called linear response theory (LRT) [20] and compute spectral quantities characterizing the neuronal spiking processes. This theory relies on the *ansatz* that the spiking response of a neuron embedded in a network can be written as the sum of the neuron’s unperturbed spiking activity—i.e., when the neuron is isolated from the fluctuations of the network activity—and its first-order response to the fluctuations of synaptic currents. Finite-size effects do not need a special treatment in that theory, but it can only manage wide-sense stationary external inputs that maintain a comfortable distance from bifurcation points. LRT has in fact been successfully used to study finite-size fluctuations in the context of decorrelative effects of inhibitory feedback [21], the interplay between correlation structures and network topology [22], and the effect of correlations on coding [23].

A more systematic approach to study finite-size effects is to construct a master equation describing the time evolution of the probability distribution of the states of the network. In that case the formalism borrows heavily from reaction kinetics in chemistry [24, 25]. The network navigates between states *via* activation and decay functions, which embody the stochastic transitions between quiescent and active spiking states. Using the master equation, one can then construct a system involving moments of the network’s activity. A troublesome feature of this approach is that lower order moments typically depend on higher order moments, thus constituting a hierarchy of moments that must be truncated in one way or the other. The chosen truncation scheme depends on the assumptions about the underlying neural activity. One possibility is to assume that the network is in an asynchronous state—defined by a constant infinite-size mean field—so that spike-spike correlations are of order 1/*N*, and a direct expansion of the moments to that order becomes possible [26–28]. In the same spirit, one can also perform a system-size expansion—to order $1/\sqrt{N}$—of the master equation, and then construct the hierarchy of moments that can be subsequently truncated [29]. Another truncation based on normal-ordered cumulants assumes near-Poisson spiking [30].

The truncation of the hierarchy of moment equations breaks down near criticality, i.e., near a bifurcation. One way to circumvent this problem is to use path integral methods [31]—borrowed from statistical field theory—which themselves are amenable to the use of renormalization group methods that can extract critical exponents near the bifurcation. These field theoretic methods have also been applied to describe the statistics of deterministic phase neurons [32]. All the approaches discussed above assume a network that is either far below criticality or near criticality.

As we see from this overview, analytical treatments are emerging and hold the expectancy of understanding the nontrivial effects of variability on neural circuits. To move toward the formulation of models that keep track of the intrinsic randomness, our challenge is to correct the usual mean-field equations to account for the inescapable stochastic nature of spike initiation. The aim of the present paper is then to take up the problem of finite-size fluctuations and to show that, actually, one can formulate it in the framework of stochastic partial differential equations (SPDE).

Contrary to other treatments of finite-population dynamics, our main objective is to describe the finite-size activity itself, and not its moments. To this end, we derive a SPDE (see Eq 7 below) that gives the activity of a finite population of spike-response model neurons with escape noise [33] for a fully connected inhibitory network. The equation describes the dynamics of the finite-size refractory density [34], i.e. the density of neurons in a given refractory state at a given time. The boundary condition (Eq 8) or the conservation law (Eq 9) for the refractory density is used to extract the activity. Finite-size fluctuations appear in the SPDE through a two-dimensional Gaussian white noise—in time and along the refractory dimension—whose prefactor vanishes in the infinite-size (thermodynamic) limit. Importantly, the Gaussian white noise acting in the network’s description naturally emerges from the intrinsic randomness of spike initiation present at the cellular level.

The SPDE can be solved numerically *via* a discretization along its characteristic curves (see Eq 55 in the Methods section), and thus provides a direct mean to simulate finite-size networks, both below and above the bifurcation towards the oscillatory state. Importantly, the simulation time does not depend on the size of the population. Bifurcation analysis of the associated mean-field counterpart enables us to reveal how delayed inhibitory feedback permits the emergence of macroscopic rhythms. More insight into finite-size effects is obtained by applying a linear noise approximation, followed by a study of the spectral and autocorrelation properties of fluctuations in the asynchronous activity regime (see Eqs 18 and 20).

Perhaps the approaches closest in spirit to the one adopted in the following are those of Meyer and van Vreeswijk [27], and Deger, Schwalger and coworkers [35, 36]. Meyer and van Vreeswijk treated finite-size fluctuations in homogeneous, fully-connected networks of renewal spike-response neurons using a refractory density approach, as in the current paper. However, their main goal was to derive equations for the temporal cross correlations in the stable stationary state. Thus, even though their framework is akin to ours, the objectives differ: instead of focusing on the moments, here we analytically build a stochastic dynamical equation that captures the temporal evolution of the activity itself, including finite-size fluctuations.

The paper by Deger, Schwalger et al. [35] dealt with the finite-size activity of randomly connected spike-response model neurons with adaptation. With their approximations, they ended up with quasi-renewal neurons [37] connected in an effective all-to-all fashion. In that context, they established an integral equation for the activity, and obtained the temporal autocorrelation function of the activity. However, this formulation makes it impossible to actually simulate the network’s activity; this is because their correlation function includes the probability density that a spike in the past causes a spike in the future, which cannot be computed in general because the future activity—which is yet unknown—is required.

In a recent work, however, Schwalger et al. [36] solved that problem by proposing an error minimizing method that permits rapid and accurate simulations of the firing statistics of the network, for a single or multiple populations. They derived stochastic differential equations that only involve the finite-size activity; contrary to our approach, information about the refractory distribution is thus purposefully disregarded. A byproduct of this approximation is that the neuron number is not conserved anymore, unlike our approach. Moreover, using a Gaussian approximation they obtained a stochastic equation—Eq 41 in their appendix—that is similar in spirit to ours, although it differs somewhat in its details. Their stochastic equation also involves the temporal evolution of the refractory distribution, but is not thoroughly analyzed.

This paper is organized as follows. First, we present the network and neuron model that will be used throughout. Then, we obtain the SPDE and perform a linear noise approximation, which is then used to study both the stability of the deterministic (mean-field) part of the activity, and the statistical properties of the finite-size fluctuations. These include spectral and autocorrelation properties.

## Results

### The neural network

We consider a fully connected (all-to-all) homogenous network of *N* neurons with inhibitory synapses. Neuronal dynamics are described using the spike-response model with escape noise [33]. For this model, neuronal spiking occurs according to an instantaneous firing intensity or hazard function that depends on the difference between the membrane potential and a fixed threshold. The membrane potential of neuron *i* at time *t* is given by

$$\begin{array}{c} \begin{array}{cc} u_{i}\left(t\right) & =-\left(V*y_{i}\right)\left(t\right)-\frac{J_{s}}{N}\sum_{j=1}^{N}\left(\kappa *y_{j}\right)\left(t\right)+\left(\kappa *I_{\text{ext}}\right)\left(t\right)\\ & \equiv -\left(V*y_{i}\right)\left(t\right)+h_{i}\left(t\right), \end{array} \end{array}$$ (1)

where *h*_*i*_(*t*) is the change in potential caused by inputs (input potential), -V is a refractory kernel representing the reset of the membrane potential following a spike, and *κ* is a filter kernel encompassing synaptic and membrane filtering of synaptic and external inputs. The spike train of any neuron is given by

$$\begin{array}{c} y_{i}\left(t\right)=\sum_{f}\delta \left(t-t_{i}^{\left(f\right)}\right), \end{array}$$

where the sum is over all of its spike times. Hence, the membrane potential of neuron *i* at time *t* is given by the convolution of its own spike train with the refractory kernel [$\left(V*y_{i}\right)\left(t\right)$], to which are added a filtered external input [(*κ* * *I*_ext_)(*t*)] and the total inhibitory synaptic input

$$\begin{array}{c} -\frac{J_{s}}{N}\sum_{j=1}^{N}\left(\kappa *y_{j}\right) \end{array}$$

coming from all neurons within the network, with uniform synaptic weight −*J*_*s*_/*N*. We shall restrict ourselves to non-adaptive single-neuron dynamics, meaning that only the most recent spike of a neuron affects its potential. Thus,

$$\begin{array}{c} \left(-V*y_{i}\right)\left(t\right)\equiv -V\left(r_{i}\right), \end{array}$$ (2)

where

$$r_{i}=t-{\hat{t}}_{i},$$

is the *refractory state* or *age* of neuron *i*, and ${\hat{t}}_{i}$ denotes its most recent spike.

We are interested in the dynamics and statistics of the population activity

$$\begin{array}{c} A\left(t\right)\equiv \frac{1}{N}\sum_{i=1}^{N}y_{i}\left(t\right). \end{array}$$ (3)

The homogeneity of the network implies that the index *i* can be dropped in Eq 1: all neurons with the same refractory state have the same subsequent dynamics. Using the definition of the population activity, Eq 1 becomes

$$\begin{array}{c} u\left(t,r\right)=\left(\kappa *\left[I_{\text{ext}}-J_{s}A\right]\right)\left(t\right)-V\left(r\right). \end{array}$$ (4)

The hazard function *ρ* is the probability per unit time of emitting a spike, conditional on the past history of the activity,

$$H_{t}\equiv \left\{A\left(t^{\prime }\right):0<t^{\prime }<t\right\},$$

and of the external signal,

$$\{I_{\text{ext}}\left(t^{\prime }\right):0<t^{\prime }<t\}.$$

**Fig 1:**
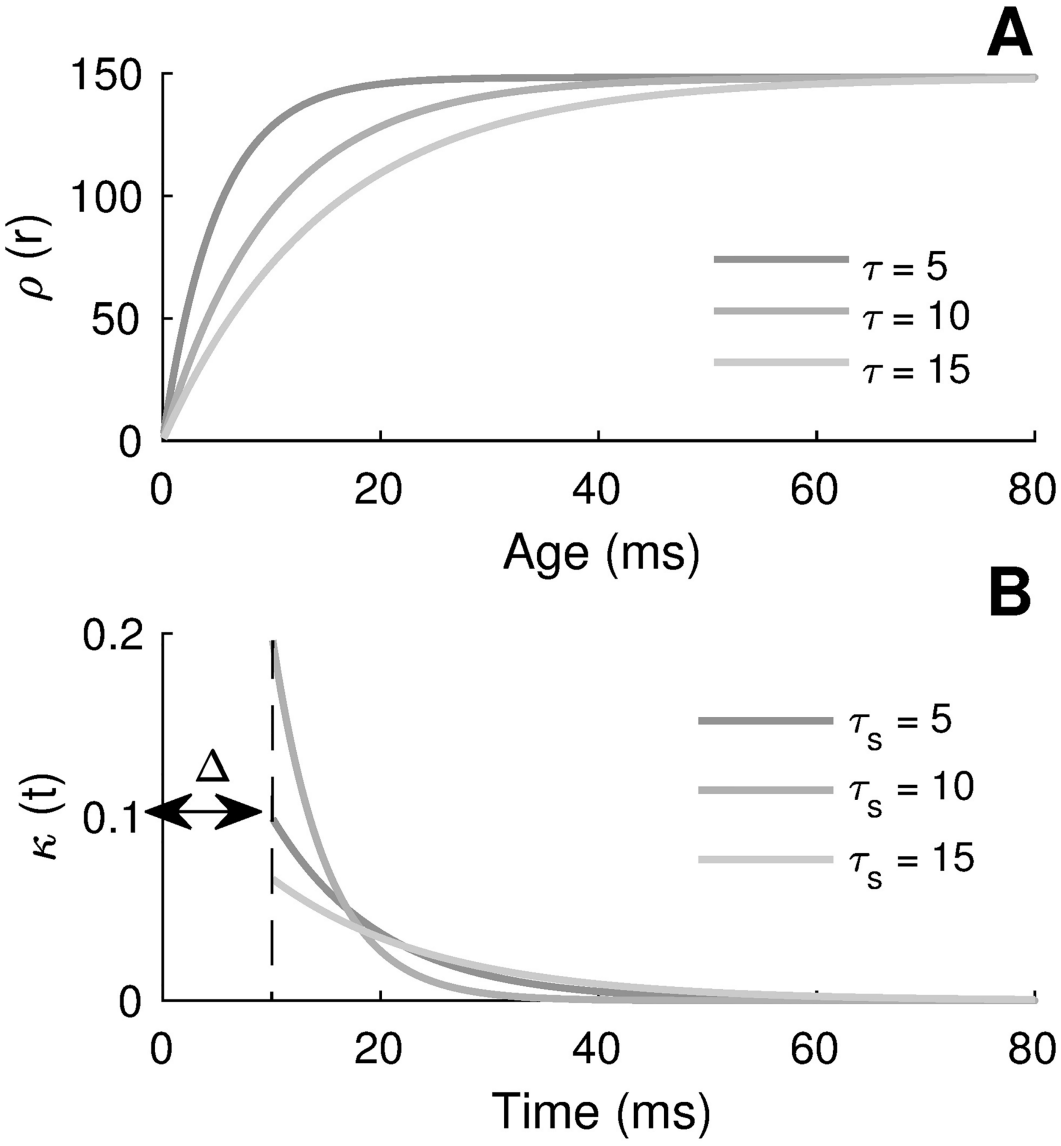
Illustration of the hazard function and the synaptic kernel. A) Hazard function *ρ*(*r*), Eq 5, when *h*(*t*) ≡ 5 and λ_0_ = 1 kHz. B) Synaptic filter *κ*(*t*) for different values of the synaptic decay *τ*_*s*_, with Δ = 10 ms (*cf.* Eq 6). The integral of *κ* over time is normalized to 1.

**Fig 2:**
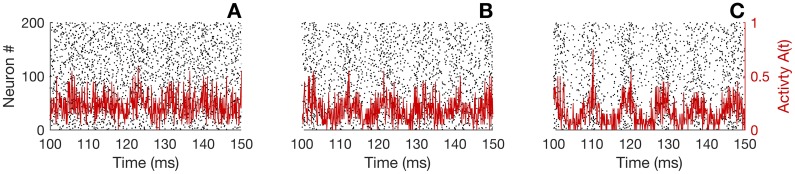
Examples of spiking activity for the neural network. The network contains *N* = 200 neurons. In each panel is shown the spiking activity of every neuron in a raster plot (dots represent spikes). The solid red line represents the activity *A*(*t*) of the network, obtained by counting the total number of spikes in a time window Δ*t* = 0.2 ms, and dividing by *N*Δ*t*. For all panels, λ_0_ = 1 kHz, *τ* = *τ*_*s*_ = 10 ms, Δ = 3 ms and *I*_ext_ = 7;. Panel A: *J*_*s*_ = 3; panel B: *J*_*s*_ = 4, panel C: *J*_*s*_ = 5.

For concreteness we will use an exponential hazard function,

$$\begin{array}{c} \rho \left[u\left(t,r\right)\right]=\lambda_{0}\exp\left[u\left(t,r\right)/\delta u\right], \end{array}$$ (5)

where λ_0_ and *δu* = 1 mV are constants. This choice has no impact on the theory presented herein, other than simplifying some computations. Also, the refractory kernel is taken to be

$$\begin{array}{c} V\left(r\right)=-\ln(1-e^{-r/\tau }), \end{array}$$

where *τ* is the recovery time scale. The synaptic filter is

$$\begin{array}{c} \kappa \left(t\right)=H\left(t-\Delta \right)\frac{e^{-\left(t-\Delta \right)/\tau_{s}}}{\tau_{s}}, \end{array}$$ (6)

with *H*(*t*) the Heaviside step function, *τ*_*s*_ the synaptic decay and Δ the conduction delay. The specific expression characterizing the escape rate is justified and largely used because it combines a relative mathematical simplicity with the capacity to realistically reproduce observed neuronal behaviors. Note that *J*_*s*_ and *I*_ext_ have units mV⋅ms and mV, respectively, because *κ* has units ms^−1^. The synaptic kernel is thus defined because in that case the average input potential is independent of the time scale *τ*_*s*_. The hazard function and the synaptic kernel are depicted in Fig 1. We also provide examples of network dynamics in Fig 2. The dynamics of every neuron follow the non-adaptive version of Eq 1 (i.e., with Eq 2), together with the escape rate firing mechanism and the hazard function of Eq 5. As *J*_*s*_ increases, the network develops an oscillatory instability (see below) and oscillations appear.

### The stochastic-field equation

The continuous deterministic mean-field approach to modeling neural networks fails to capture many important details. The missing detail manifests itself as small unpredictable finite size fluctuations present at the network level. Our main challenge is then to define an equation that fully entails the stochastic aspects of the network. To do so, we consider the finite-size refractory density *q*(*t*, *r*), such that *Nq*(*t*, *r*)*dr* gives the number of neurons with age in [*r* − *dr*, *r*) at time *t*. A time *dt* later, all neurons that did not fire will have aged by an amount *dr* = *dt*, whereas the number of neurons that did fire is given by a Poisson random variable P with rate *Nρ*(*t*, *r*)*q*(*t*, *r*)*dtdr* [25, 35]. This idea encompasses the presence of fluctuations that are proportional to the mean number of firing events and therefore retains the full random character of the spiking probability. Using a Gaussian approximation of this Poisson distribution, i.e. making the approximation

$$\begin{array}{c} P(N\rho (t,r)q(t,r)dtdr)∼N\rho \left(t,r\right)q\left(t,r\right)dtdr+\sqrt{N\rho (t,r)q(t,r)dtdr}N\left(0,1\right), \end{array}$$

where N(0,1) denotes the standard normal distribution and ∼ means “is distributed like”, we show (see Methods) that *q*(*t*, *r*) obeys the following stochastic partial differential equation (SPDE):

$$\begin{array}{c} \begin{array}{c} \frac{\partial }{\partial t}q\left(t,r\right)+\frac{\partial }{\partial r}q\left(t,r\right)=-\rho \left(t,r\right)q\left(t,r\right)-\sqrt{\frac{\rho (t,r)q(t,r)}{N}}\eta \left(t,r\right), \end{array} \end{array}$$ (7)

where *η* is a Gaussian (sheet) white noise with

$$\begin{array}{c} \left\langle \eta \left(t,r\right)\right\rangle =0,\;\left\langle \eta \left(t,r\right)\eta \left(t^{\prime },r^{\prime }\right)\right\rangle =\delta \left(t-t^{\prime }\right)\delta \left(r-r^{\prime }\right), \end{array}$$

and

$$\begin{array}{c} \rho \left(t,r\right)=\lambda_{0}\exp\left[u\left(t,r\right)\right]. \end{array}$$

The brackets denote an ensemble average over realizations of the stochastic process. The boundary condition is naturally given by the reset mechanism. Indeed, once a neuron triggers a spike, its age is reset to zero. Therefore, the boundary condition is

$$\begin{array}{c} q(t,0)=A(t), \end{array}$$ (8)

where *A*(*t*) is the finite-size activity of the network (see for instance Fig 2). This activity *A*(*t*) is also given by the total rate at which neurons of all ages escape their trajectories in the (*t*, *r*)-plane:

$$\begin{array}{c} A\left(t\right)=\int_{0}^{\infty }\rho \left(t,r\right)q\left(t,r\right)\;dr+\int_{0}^{\infty }\sqrt{\frac{\rho (t,r)q(t,r)}{N}}\eta \left(t,r\right)\;dr. \end{array}$$ (9)

The integral above is to be understood in the Itô sense. Finally, the refractory density must obey a conservation law,

$$\begin{array}{c} \int_{0}^{\infty }q\left(t,r\right)\;dr=1, \end{array}$$ (10)

since each neuron possesses a given age at any given time. Note that both *q*(*t*, *r*) and *A*(*t*) are stochastic processes in this formulation.

**Fig 3:**
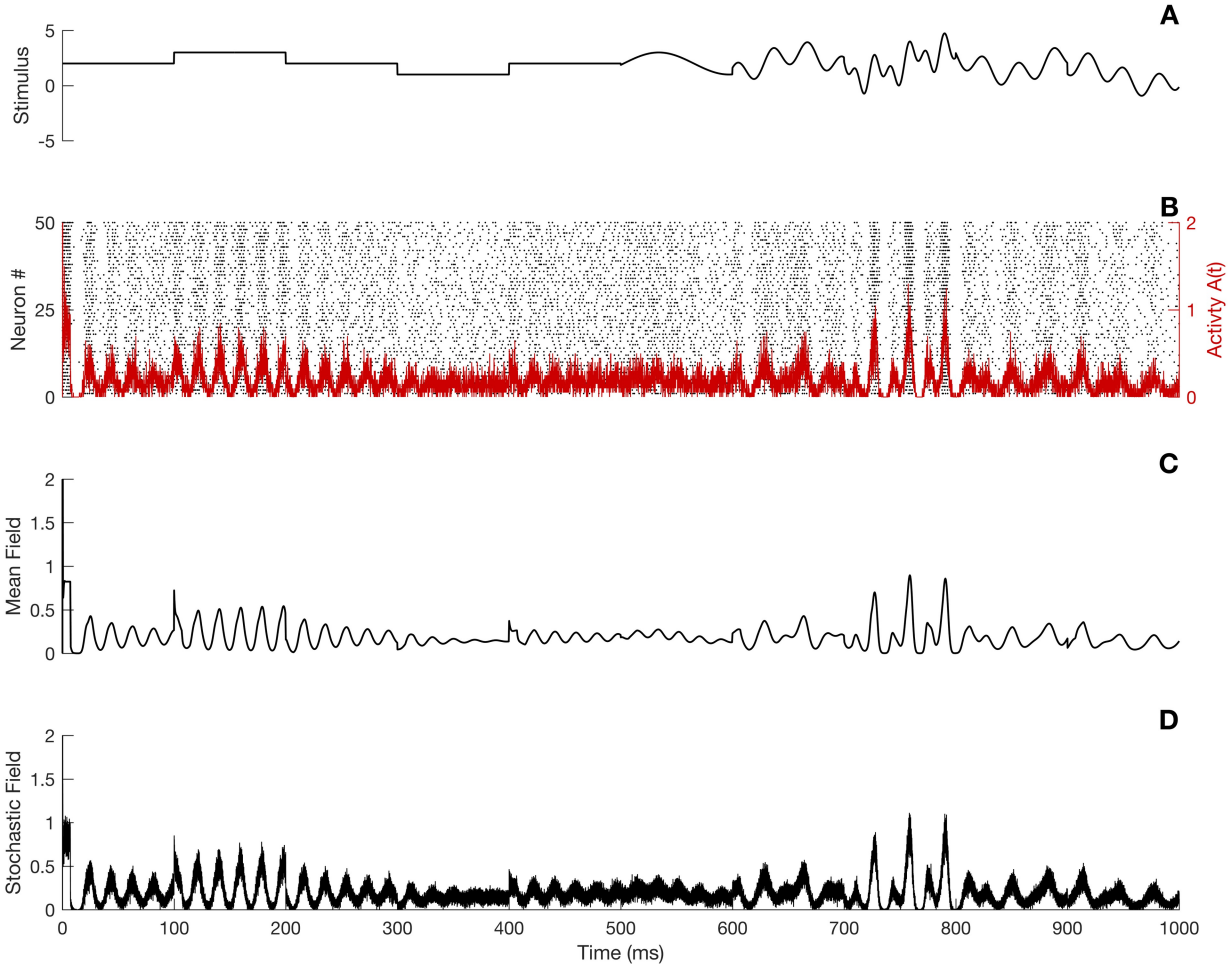
Simulation of the SPDE and comparison with the full network dynamics and the mean-field theory for a time dependent stimulus. A) Time evolution of the stimulus. B) Activity obtained from simulations of the full network. C) Activity obtained from simulations of the mean-field equation (Eq 13). D) Activity obtained from the SPDE (Eq 7). The same initial condition was used in all cases. Parameters are as in Fig 2C, except that *N* = 500 and Δ*t* = 0.1 ms.

**Fig 4:**
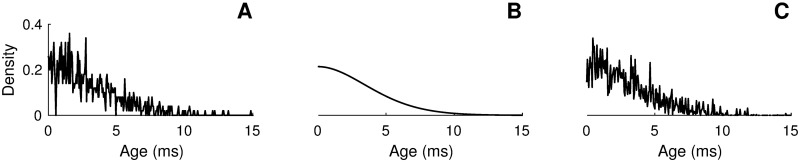
Refractory density *q*(*t*, *r*) as computed from A) the full network, B) the mean-field limit (Eq 13) and C) the SPDE (Eq 7). The same initial condition was used in all cases. Parameters are as in Fig 2A, except Δ*t* = 0.1 ms, *t* = 1000.

From the derivation above we observe that for a network with *N* cells, the relative magnitude of fluctuations scale as $1/\sqrt{N}$. The stochastic parts in Eq 7 as well as in Eq 9 disappear in the thermodynamic limit when the network is taken infinitely large. Doing so, the equation finally reduces to the classical refractory equation [33]. However, the thermodynamic limit does not allow for any characterization of fluctuations around the mean activity, because it is an entirely deterministic approach as is illustrated in Figs 3 and 4.

Fig 3A shows the time evolution of the external stimulus, whereas panel B gives the spiking activity obtained from a simulation of the full network and its corresponding activity. The activity given by the standard mean field in the thermodynamic limit (*N* taken infinitely large, see Eq 13 below) is shown in Fig 3C. Although in this case the mean-field approximation captures the essential “shape” of the activity of the full network shown in the panel B, it completely ignores the substantial finite-size fluctuations. Finally, Fig 3D shows the activity as generated via a simulation of the stochastic-field equation (Eq 7). The stochastic-field equation effectively captures both the shape and variability of the full neural activity, which could be described as a noisy version of the mean-field activity. Similar observations can be made regarding the refractory density *q*(*t*, *r*) as can be seen in Fig 4. The numerical integration of Eq 7 is discussed in the Methods section.

Note that multiple populations can be modeled straightforwardly using the above formalism. To each population *n* would be assigned a refractory density *q*_*n*_(*t*, *r*) obeying the SPDE. The respective membrane potentials would be given by

$$\begin{array}{c} u_{n}\left(t,r\right)=\left(\kappa_{n}*\left[I_{\text{ext}}-\sum_{k}J_{nk}A_{k}\right]\right)\left(t\right)-V_{n}\left(r\right) \end{array}$$

with *J*_*nk*_ the total synaptic strength connecting population *k* to population *n*. Different hazard functions *ρ*_*n*_ can be chosen as well.

An analytical solution of the SPDE is exceedingly difficult, if not impossible. However, informations about the statistics of activity fluctuations can be extracted *via* a system-size expansion, as discussed in the next section.

### Linear noise approximation

To investigate the effects of fluctuations for large but finite network size *N*, we perform a linear noise approximation (LNA) when *I*_ext_ is constant. In our situation, the LNA states that the density function as well as the neural activity can be approximated by the sum of a deterministic and a stochastic process. The fluctuating part is scaled by a $1/\sqrt{N}$ factor that is justified by the Van Kampen system-size expansion [24]. This system-size expansion is usually pursued only up to first order, hence the “linear” qualifier. We write

$$\begin{array}{c} q\left(t,r\right)=q_{0}\left(t,r\right)+\frac{1}{\sqrt{N}}q_{\xi }\left(t,r\right)+O(\frac{1}{N}), \end{array}$$ (11)

with *q*_0_(*t*, *r*) and *q*_*ξ*_(*t*, *r*) the deterministic and stochastic parts, respectively. Similarly, the population activity reads

$$\begin{array}{c} A\left(t\right)=A_{0}\left(t\right)+\frac{1}{\sqrt{N}}A_{\xi }\left(t\right)+O(\frac{1}{N}), \end{array}$$ (12)

again with *A*_0_(*t*) the deterministic part and *A*_*ξ*_(*t*) the stochastic part. After algebraic manipulations—including a linearization that only keeps the first-order term in $1/\sqrt{N}$—we find that the deterministic part obeys the usual mean-field description

$$\begin{array}{c} \frac{\partial }{\partial t}q_{0}\left(t,r\right)+\frac{\partial }{\partial r}q_{0}\left(t,r\right)=-\rho_{0}\left(t,r\right)q_{0}\left(t,r\right) \end{array}$$ (13)

with boundary condition

$$\begin{array}{c} q_{0}\left(t,0\right)=A_{0}\left(t\right), \end{array}$$

where *A*_0_(*t*) is the deterministic part of the activity. The hazard *ρ*_0_(*t*, *r*) is

$$\begin{array}{c} \rho_{0}\left(t,r\right)\equiv \rho \left[u_{0}\left(t,r\right)\right], \end{array}$$

with

$$\begin{array}{c} u_{0}\left(t,r\right)\equiv \left(\kappa *\left[I_{\text{ext}}-J_{s}A_{0}\right]\right)\left(t\right)-V\left(r\right). \end{array}$$

Note that the mean field equation is strikingly similar to the standard age-structured system known as the McKendrick von-Foerster model in mathematical biology [38, 39].

The stochastic component solves a SPDE similar to Eq 7, namely (see Methods)

$$\begin{array}{r} \frac{\partial }{\partial t}q_{\xi }\left(t,r\right)+\frac{\partial }{\partial r}q_{\xi }\left(t,r\right)=J_{s}\left(\kappa *A_{\xi }\right)\left(t\right)\frac{d\rho }{du}{|}_{u_{0}}\;q_{0}\left(t,r\right)-\sqrt{\rho_{0}\left(t,r\right)q_{0}\left(t,r\right)}\eta \left(t,r\right)\\ -\rho_{0}\left(t,r\right)q_{\xi }\left(t,r\right) \end{array}$$ (14)

and the derivative ${d\rho /du|}_{u_{0}}$ is evaluated at *u*_0_. This equation is now linear in the function *q*_*ξ*_—contrary to Eq 7—and therefore an analytical solution is possible (this is done in Methods in the context of a computation of the power spectrum of activity fluctuations).

**Fig 5:**
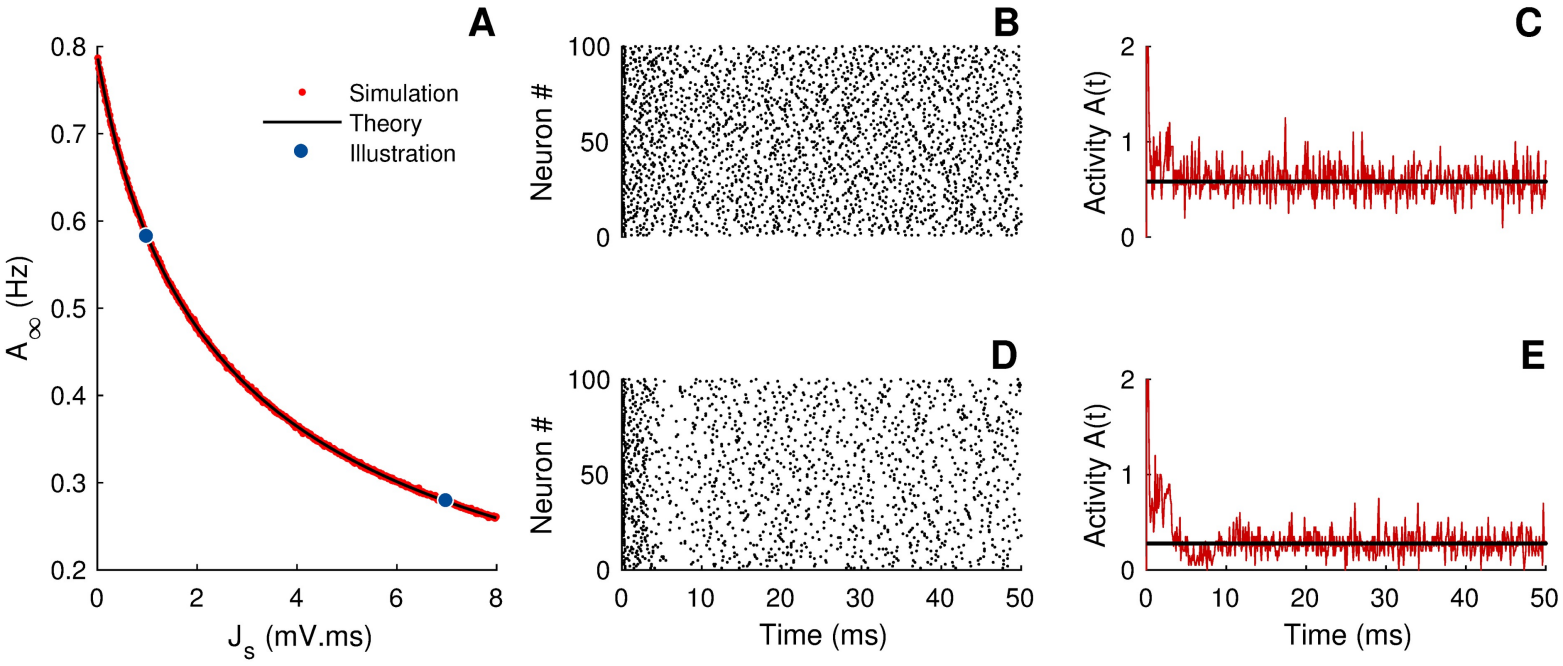
Relaxation towards the steady state activity. A) Steady state activity in the asynchronous regime as a function of the synaptic coupling *J*_*s*_. Since *J*_*s*_ actually represents the strength of inhibition, *A*_∞_ decreases when *J*_*s*_ increases. The black curve is obtained by computing *A*_∞_ from Eq 15. The small red points and the two large blue dots are steady-state activities computed from simulations of the stochastic neural network. B) and D) The spiking activity is depicted as raster plots. The two simulations correspond to the parameters given by the two large blue dots in panel A. C) and E) Comparison between the neural activity of the fully stochastic neural network (red curve) and the deterministic activity (constant black line) obtained by solving Eq 15. Parameters were *N* = 100, *I*_ext_ = 2 mV, *τ* = 7 ms, *τ*_*s*_ = 5 ms, Δ = 3 ms and λ_0_ = 1 kHz. The discretization time step for computing the activity (red curves) was Δ*t* = 0.2 ms.

When the deterministic component exhibits multiple stable states, the LNA fails to capture transitions between these states. While the LNA is inadequate for this type of situation, it is pertinent when the network fluctuates around a unique stable equilibrium, as illustrated in Fig 5. The stochastic activity is given to first order by Eq 12. After a short transient (∼10 ms), the deterministic part of the activity, *A*_0_(*t*), asymptotically reaches its steady state value *A*_∞_ (see black curve in Fig 5C and 5E). Since the neuron number is finite, fluctuations around the deterministic activity are observed, both in the transient and steady states (red curve Fig 5C and 5E). As illustrated in panels B and D of this figure, the activity of the relaxed network is asynchronous.

The average activity in the asynchronous state, *A*_∞_, can be computed as the time-independent solution of the infinite-size refractory density equation, Eq 13. This means that the partial derivative with respect to time is zero. After algebraic manipulations, we find that *A*_∞_ is given by (see Methods)

$$\begin{array}{c} \begin{array}{c} A_{\infty }^{-1}=\int_{0}^{\infty }e^{-\int_{0}^{r}\rho_{\infty }\left(s\right)\;ds}\;dr, \end{array} \end{array}$$ (15)

where

$$\begin{array}{c} \rho_{\infty }\left(s\right)\equiv \rho \left[u_{\infty }\left(s\right)\right]=\rho \left[h_{\infty }-V\left(s\right)\right], \end{array}$$

and

$$\begin{array}{c} h_{\infty }=I_{\text{ext}}-J_{s}A_{\infty }. \end{array}$$

The equation above must be solved self-consistently for *A*_∞_. From Fig 5A, the agreement between analytical and numerical results is excellent.

### Deterministic oscillatory instability

**Fig 6:**
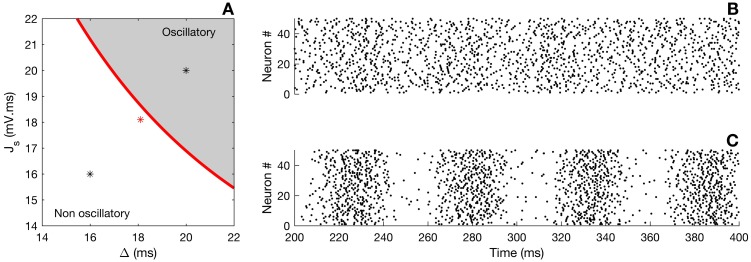
Oscillatory instability. If the synaptic strength *J*_*s*_ and the conduction delay Δ are large enough, then the system undergoes a Hopf bifurcation. A) Bifurcation line in parameter space (red curve). The grey shaded region corresponds to an oscillatory regime of the neural network. B) and C) Raster plots of the spiking activity for 50 cells. Panel B corresponds to the black asterisk lying in the asynchronous (white) region in panel A, whereas panel C depicts the activity in the oscillatory state. Parameters were *I*_ext_ = 2 mV⋅ms, λ_0_ = 1 kHz, *τ* = 10 ms and *τ*_*s*_ = 10 ms.

The asynchronous state can lose stability through an oscillatory instability. Finite-size fluctuations will potentially influence the presence of oscillations. To address this issue, we performed a linearization of the deterministic field equation (Eq 13) around the steady state (see Methods). This allowed us to write the characteristic equation, whose solutions give the eigenvalues λ of the deterministic system. The characteristic equation is denoted

$$\begin{array}{c} C(\lambda )=0, \end{array}$$

with

$$\begin{array}{ll} C\left(\lambda \right)\equiv & 1-{\hat{P}}_{\infty }\left(\lambda \right)-J_{s}\hat{\kappa }\left(\lambda \right)A_{\infty }\int_{0}^{\infty }dr\int_{0}^{r}ds\;P_{\infty }\left(r\right){\frac{d\rho }{du}|}_{u_{\infty }\left(s\right)}e^{-\lambda \left(r-s\right)}\\ & +J_{s}\hat{\kappa }\left(\lambda \right)\int_{0}^{\infty }{\frac{d\rho }{du}|}_{u_{\infty }\left(r\right)}q_{\infty }\left(r\right)dr. \end{array}$$ (16)

where $\hat{\kappa }\left(\lambda \right)$ is the Laplace transform of *κ* and *P*_∞_(*r*) is the steady-state interspike interval probability density of the asynchronous infinite-size network, i.e. the probability density of obtaining an interval

$$\begin{array}{c} r=t-\hat{t}, \end{array}$$

see Eq 46. Also,

$$\begin{array}{c} q_{\infty }\left(r\right)=\lim_{t\to \infty }q_{0}\left(t,r\right) \end{array}$$

is the steady state of the infinite-size refractory density. The eigenvalues are thus given by the roots of C(λ). The time-independent solution *A*_∞_ will be stable if all eigenvalues have negative real parts. The bifurcation line—which separates the oscillatory regime from the asynchronous one—can be drawn numerically, as depicted in Fig 6A. The red line constitutes the boundary between the stability and the instability regions for the chosen *I*_ext_. The shaded area defines the region in parameter space where self-sustained oscillations are going to emerge. Well below the transition line, in the asynchronous regime, damped oscillations may occur; however, the network activity will eventually settle into finite-size fluctuations around a constant mean activity. Above the transition, network oscillations are clearly noticeable (Fig 6C).

The existence of oscillations in fully connected, delayed inhibitory networks is well known [40], and underlies the ING (Interneuron Network Gamma) mechanism for generating gamma rhythms (see for instance [41]). The above analysis serves the purpose of delineating the oscillatory and asynchronous states in the phase diagram of Fig 6 with the help of the characteristic function. Both the phase diagram and the characteristic function will come handy in the next section where we will study the fluctuations of the asynchronous state. Note, however, that we shall not study the exact nature of the bifurcation; for the network studied in [40], the Hopf bifurcation can be either subcritical or supercritical, depending on the inhibitory coupling strength.

### Fluctuations in the asynchronous regime

Asynchronous firing has been repeatedly associated with the background activity in cortical networks [42]. Finite-size effects generate fluctuations whose basic statistics are closely related to the response of the associated infinite-size dynamics. To characterize the statistical content of fluctuations we compute the power spectrum of the activity in the asynchronous regime. The power spectrum describes how the variance of *A*(*t*) is distributed over the frequency domain and accordingly helps to identify the dominant frequencies, if any.

**Fig 7:**
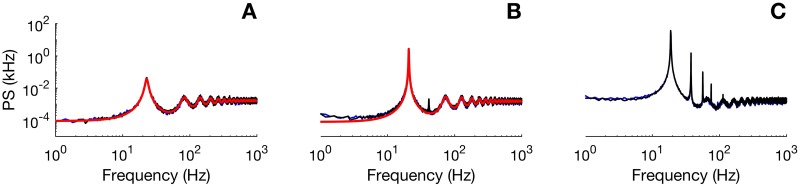
Power spectrum (PS) of finite-size fluctuations of the network activity *A*(*t*) as a function of frequency *f* = *ω*/2*π*. We display the theoretical PS (solid red curve), omitting the DC component at *ω* = 0 (*cf*. Eqs 17 and 18), as well as an average spectrum (black trace) over 50 realizations of the SPDE. The blue curve is the PS of the full spiking network activity. Parameters were *I*_ext_ = 2 mV⋅ms, λ_0_ = 1 kHz, *τ* = 10 ms and *τ*_*s*_ = 10 ms. A-B-C correspond to the three stars of Fig 6A.

By definition, the power spectrum is given by

$$\begin{array}{c} P\left(\omega \right)=\underset{T\to \infty }{\text{lim}}\frac{\langle {\left|{\tilde{A}}_{T}\left(\omega \right)\right|}^{2}\rangle }{T}, \end{array}$$

where

$$\begin{array}{c} {\tilde{A}}_{T}\left(\omega \right)=\int_{0}^{T}A\left(t\right)e^{-i\omega t}\;dt \end{array}$$

is the Fourier transform of the neural activity restricted to a time interval and the brackets 〈⋅〉 denote an average over the noisy realizations of the network dynamics. To derive an analytical expression for this quantity, we assume that the deterministic part of the activity has reached its equilibrium state (below the threshold of instability). This means that Eq 12 becomes

$$\begin{array}{c} A\left(t\right)=A_{\infty }+\frac{1}{\sqrt{N}}A_{\xi }\left(t\right), \end{array}$$

neglecting terms of order 1/*N* and above. Hence,

$$\begin{array}{c} P\left(\omega \right)=2\pi A_{\infty }^{2}\delta \left(\omega \right)+\frac{P_{\xi }\left(\omega \right)}{N}, \end{array}$$ (17)

with $P_{\xi }\left(\omega \right)/N$ the power spectrum of the fluctuations. In Methods, $P_{\xi }\left(\omega \right)$ is shown to be

$$\begin{array}{c} \begin{array}{cc} P_{\xi }\left(\omega \right) & =A_{\infty }\frac{1+\int_{0}^{\infty }ds\;\rho_{\infty }\left(s\right)e^{\int_{0}^{s}\rho_{\infty }\left(x\right)dx}{\left|\int_{s}^{\infty }dre^{-i\omega r}P_{\infty }\left(r\right)\right|}^{2}}{{\left|C\left(i\omega \right)\right|}^{2}}\\ & -\frac{2A_{\infty }ℜ\left\{\int_{0}^{\infty }ds\int_{0}^{s}dr\;\rho_{\infty }\left(s\right)P_{\infty }\left(r\right)e^{-i\omega \left(r-s\right)}\right\}}{{\left|C\left(i\omega \right)\right|}^{2}}. \end{array} \end{array}$$ (18)

where ℜ means to take the real part of the expression in curly brackets and *P*_∞_(*r*) is the interspike interval distribution in the asynchronous state. The stability of the deterministic system—embodied in its characteristic equation C(λ)=0—appears explicitly in the above expression. Of course, in the asynchronous regime there are no pure imaginary solutions to this equation. However, the minima of C(iω) dictate the position of dominant frequencies, as illustrated in Fig 7. This figure also compares the power spectrum obtained from Eq 18 to the power spectrum obtained from both an average over different realizations of the fully stochastic neural network and the SPDE; it shows an excellent agreement. We carried out the comparison for more simulations than are shown here, and our results hold over a wide range of parameters.

We note that there is a discrepancy between theory and numerics mainly at lower frequencies as the bifurcation is approached from below (Fig 7B). The theoretical power spectrum of Eq 18 does not capture the emergence of harmonics (for instance, the small peak around *f* = 400 Hz in Fig 7B). The LNA is known to fail as soon as parameters are chosen to be close to a bifurcation point. The spectra computed from the SPDE agree very well with the spectra from the whole network for all the regimes shown. Thus the SPDE can be used to provide a good picture of a spiking network’s spectral properties without having to resort to the much longer full network simulations, at least when the network size is not too small.

**Fig 8:**
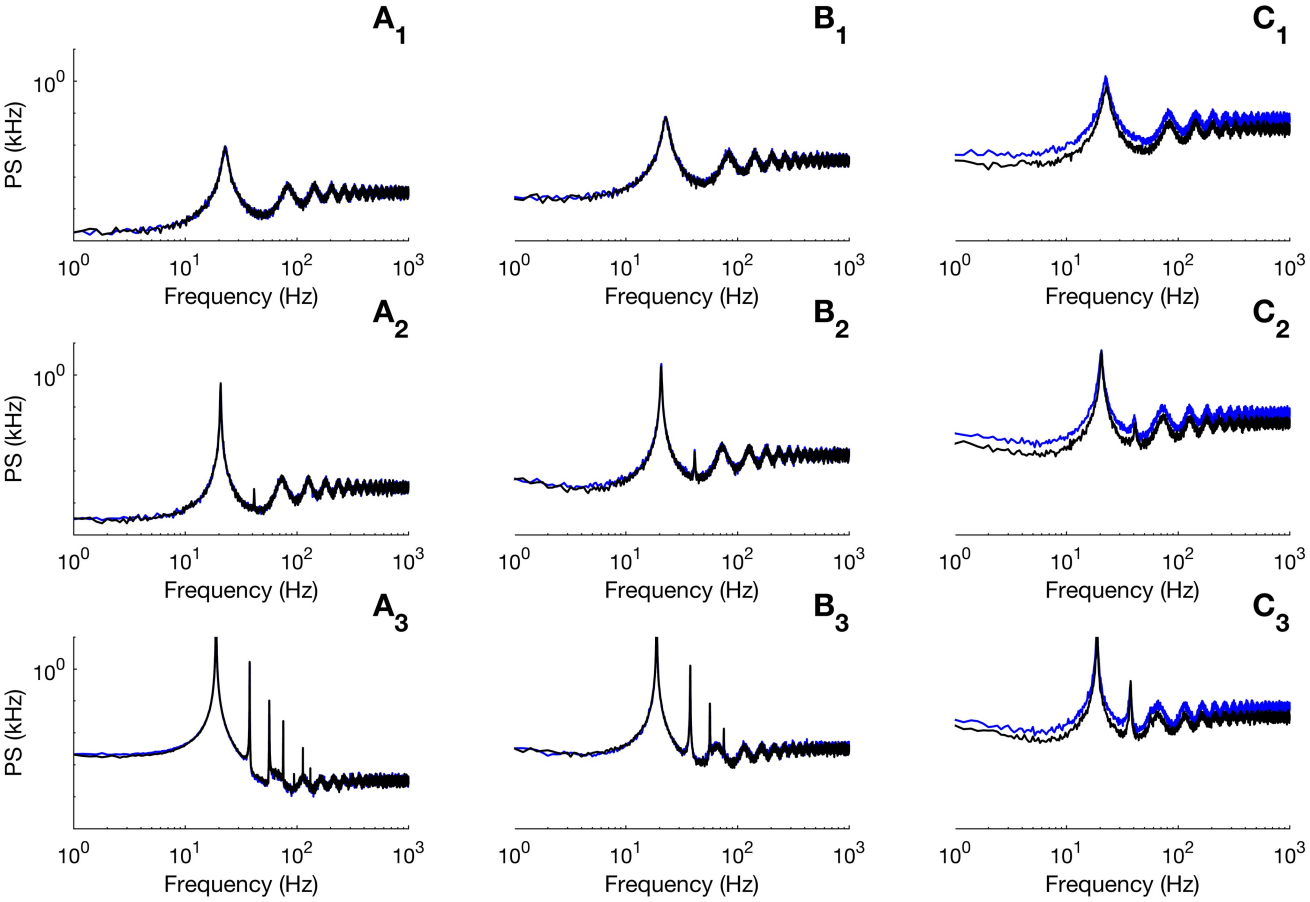
Discrepancy between the SPDE and the full network for small network sizes. We display the average spectrum over 50 realizations of the SPDE (black traces) and of the full spiking network (blue traces). Panels A, B and C correspond to simulations with network sizes *N* = 1000, 100 and 10, respectively. The subscripts 1 to 3 correspond to the three stars in Fig 6A: number 1 corresponds to the star in the non-oscillatory regime, 2 represents the red star and 3 corresponds to the rightmost star in the oscillatory regime. Other parameters as in Fig 6.

Another important remark concerns the discrepancy between the SPDE and the full network for very small system sizes. To illustrate this effect, we show in Fig 8 comparisons between the power spectrum of the full network and the SPDE for different sizes. The simulations presented in Fig 8 correspond to parameters under the bifurcation line (top row), close to the bifurcation line (middle row), and above the bifurcation (bottom row). The chosen parameter values within these regimes correspond to the three stars of Fig 6A. Panels A, B and C are illustrations for different number of cells. We clearly see that the SPDE gives very good results for large *N*, for all regimes. However, discrepancies appear when the number of cells becomes too small (of order ∼10; panels C). This is no surprise since our theory is expected to fail for very small networks. Indeed, the Gaussian approximation to the Poisson variable (see Methods) that is used to arrive at the SPDE (Eq 7) implicitly assumes the number of cells to be large enough. Hence the observed discrepancy when the number of cells is too small.

Another way to obtain the power spectrum would be to first compute the autocorrelation function of *A*_*ξ*_(*t*) and then use the Wiener-Khintchin theorem. However, solving Eq 14 provides a self-consistent expression (see Methods), namely

$$\begin{array}{c} A_{\xi }\left(t\right)=\left(Q*A_{\xi }\right)\left(t\right)+\Sigma \left(t\right). \end{array}$$ (19)

Here, *Q* represents the response to finite-size perturbations of the activity in the past, whereas Σ gives the effect of the Gaussian noise *η*(*t*, *r*) on the stochastic part of the activity (see Eq 54 and following in Methods for expressions for *Q* and Σ). Eq 19 can be solved by taking the Fourier transform, thus forcing us to first compute the power spectrum to get the autocorrelation function. Interestingly, however, the autocorrelation function of Σ(*t*) can be obtained directly in terms of the interspike interval distribution in the asynchronous state, giving (see Methods)

$$\begin{array}{c} \left\langle \Sigma \left(t\right)\Sigma \left(t+s\right)\right\rangle =A_{\infty }\delta \left(s\right)-A_{\infty }\int_{0}^{\infty }dr\;P_{\infty }\left(r\right)P_{\infty }\left(r+|s|\right), \end{array}$$ (20)

which corresponds to that obtained in [35].

## Discussion

The origin and functional implications of variability at the network scale are ongoing questions of interest in neuroscience. There have been a number of earlier efforts to go beyond the mean-field approximation to address these questions. In the past few years the idea of studying spiking neural circuit within the framework of statistical physics to investigate finite-size effects has become a concrete research project (see e.g. [43]). In this study, an alternative method was proposed to adequately describe observable noise at the network level.

We derived a stochastic partial differential equation (SPDE) describing the dynamics of the refractory density *q*(*t*, *r*) (Eq 7). This quantity gives the density of neurons in refractory state *r* at a certain time *t*, the refractory state being the time interval from the last spiking event. The population activity *A*(*t*) can be extracted, for instance, from the boundary condition *q*(*t*, 0). In the limit where the neuron number *N* goes to infinity, the standard PDE for the infinite-size refractory density is recovered.

An important point about our derivation is that we did not assume that the single-neuron spiking processes were described by a (inhomogeneous) Poisson process. In particular, as per renewal theory, the firing intensity (or hazard function) depended on the last spike, contrary to Poisson processes. Poisson random variables appeared in the course of the derivation because the number of neurons firing in a small time interval Δ*t* is a random variable following a binomial distribution, which becomes a Poisson distribution in the limit Δ*t* goes to zero. From there, the only assumption to arrive at Eq 7 was to approximate this Poisson distribution by a Gaussian distribution—the Gaussian approximation. This is in contrast with the work of Brunel and Hakim [18, 44], which starts with deterministic single-neuron membrane equations, but approximates the spiking process of neurons embedded in the network by a Poisson process. Nonetheless, the two approaches lead to a similar description, where the number of spikes produced during a small amount of time can be approximated by a Gaussian random variable.

Our SPDE cannot be solved analytically since the stochastic term involves a square-root nonlinearity. Nonetheless, a discretization along the characteristic curves of the SPDE offers a numerical scheme that gives a very satisfying approximation to its behavior. The advantage over numerical simulations of the full network is that it overcomes some of the restrictions imposed by computation time in multiple neuron simulations. Indeed, the numerical simulation of the SPDE is independent of the number of neurons. Therefore the stochastic-field density approach appears to be a viable method to make simulations of large neural networks all the while keeping track of their intrinsic variability.

On the other hand, a system-size expansion restricted to first order in $1/\sqrt{N}$—the linear noise approximation—gives rise to two coupled linear SPDEs which are amenable to analytical investigations. In particular, we studied finite-size fluctuations in the asynchronous regime, for which the average network activity is constant and neurons fire asynchronously. This enabled an analytical expression for the power spectrum of these fluctuations to be obtained (Eq 18). Its structure is determined both by the characteristic function of the deterministic system (thermodynamic limit) and by the spiking properties of the network (e.g., the interspike interval density and the hazard function). The characteristic function appears in the stability analysis of the deterministic system: zeros of this function are eigenmodes of the deterministic dynamics. Similarly, *via* an integration along the characteristic curves, some salient aspects of the autocorrelation properties of the finite-size fluctuations in the asynchronous activity regime were computed (Eq 20), allowing comparison with the literature. We therefore note that our SPDE permits the analytical computations of both the spectral Eq (18) and the correlation Eq (20) properties of the fluctuations.

We restricted our study to fully connected networks of non-adaptive (renewal) neurons. However, it should be noted that it is possible to take care of non-renewal aspects of neural dynamics using the approximation provided by the quasi-renewal theory [37]. That theory suggests that one can replace the full adaptation potential (Eq 1) by the sum of a contribution from the last spike, -V(r), and an average potential caused by other spikes over the whole past. This latter contribution can be formulated as a convolution of the population activity with a kernel. Thus, it can simply be included in an extended version of the SPDE formalism presented here.

The stochastic-field approach to finite-size effects in neural network dynamics presented herein uses a surrogate quantity *q*(*t*, *r*) to access the population activity. With this approach, finite-size noise explicitly appears in the main equation, Eq 7, taking the form of a Gaussian white noise in the variables *t* and *r*, divided by $\sqrt{N}$. This equation can be integrated numerically, providing information about the latent, refractory state of neurons. This information is typically not present in other approaches that deal with finite-size effects. Moreover, and most importantly, the firing activity can thus be directly simulated by using the SPDE; such direct simulation is typically not possible with other methods (but see below). On the other hand, the linear noise approximation gives analytical expressions for the power spectrum of finite-size activity fluctuations in the asynchronous state, a feat also possible by other approaches. For instance, the linear response theory applied to leaky integrate-and-fire networks readily generates this power spectrum [20]. Also, an integral equation has recently been proposed that directly involves the population activity [35]; linearizing this equation yields the power spectrum. Our preliminary investigations and Ref. [45] suggest that all these approaches lead to analogous results.

Moreover, Schwalger *et al.* recently used an error minimizing procedure to directly describe the activity of finite-size networks at a so-called mesoscopic level. This procedure *de facto* sacrifices probability conservation to obtain a nevertheless accurate activity equation in the time domain. Our approach rather involves both time and refractoriness, which is still considered the microscopic level by these authors. However, working directly with our SPDE provides some advantages. First, even though we performed a Gaussian approximation of the Poisson spiking statistics, the probability is still conserved, which is the standard expectation for biophysically interpreted neural mass models [14]. Second, as stated above, its linearization in combination with the use of the method of characteristics enabled us to obtain a stochastic differential equation—along the characteristics—and to compute spectral and correlation properties of the network activity. Finally, we note that our approach can also be extended to multiple populations, and we pointed the reader in the right direction for doing so without performing the full calculations.

## Methods

### Derivation of the stochastic partial differential equation

We consider a homogeneous neural population and we denote the density of neurons in refractory state *r* at time *t* by *q*(*t*, *r*). The number of neurons with their refractory state in [*r* − Δ*r*, *r*) at time *t*, denoted *m*(*t*, *r*), is given by

$$\begin{array}{c} m\left(t,r\right)\equiv N\int_{r-\Delta r}^{r}q\left(t,x\right)dx. \end{array}$$ (21)

At time *t*, a given neuron is in refractory state *r* if its last spike occurred at

$$\begin{array}{c} \hat{t}=t-r, \end{array}$$

and survived without spiking until *t*. Accordingly, *m*(*t*, *r*) represents the number of neurons a time *t* whose last spike $\hat{t}$ belongs to interval (*t* − *r*, *t* − *r* + Δ*r*]. By extension, *m*(*t* + Δ*t*, *r* + Δ*r*) represents the number of neurons whose last spike also occurred in (*t* − *r*, *t* − *r* + Δ*r*], but that survived without spiking until *t* + Δ*t*. Intuitively, *m*(*t* + Δ*t*, *r* + Δ*r*) is equal to *m*(*t*, *r*) minus the number of these neurons that did spike in [*t*, *t* + Δ*t*). If Δ*t* is small enough—so that the hazard function *ρ*(*t*, *r*) is nearly constant on this interval—then this number is a statistically independent Poisson random variable with mean and variance equal to *ρ*(*t*, *r*)*m*(*t*, *r*)Δ*t* [25, 35]. Therefore,

$$\begin{array}{c} m(t+\Delta t,r+\Delta r)\approx m(t,r)-P\{\rho (t,r)m(t,r)\Delta t\}, \end{array}$$ (22)

where P is the aforementioned Poisson-distributed random number.

Assuming that

$$\begin{array}{c} \rho (t,r)m(t,r)\Delta t\gg 1, \end{array}$$

then the Poisson distribution from which samples are randomly drawn approaches a normal distribution. In this case, we can write

$$\begin{array}{c} P\left\{\rho \left(t,r\right)m\left(t,r\right)\Delta t\right\}\approx \rho \left(t,r\right)m\left(t,r\right)\Delta t+\sqrt{\rho (t,r)m(t,r)\Delta t}N\left(0,1\right), \end{array}$$ (23)

where N(0,1) is a standard normal random variable. On the discretized (*t*, *r*)-space implicitly defined above, there exists one such standard normal random variable for every point of that space; these random variables are mutually independent.

Replacing Eq 21 in Eq 22 and using Eq 23 yields

$$\begin{array}{ll} \int_{r}^{r+\Delta r}q\left(t+\Delta t,x\right)dx-\int_{r-\Delta r}^{r}q\left(t,x\right)dx= & -\rho \left(t,r\right)\Delta t\int_{r-\Delta r}^{r}q\left(t,x\right)dx\\ & -\sqrt{\frac{\rho \left(t,r\right)\int_{r-\Delta r}^{r}q\left(t,x\right)dx\Delta t}{N}}N\left(0,1\right) \end{array}$$

After a change of integration variable the left-hand side becomes

$$\begin{array}{cc} \int_{r-\Delta r}^{r}\left[q\left(t+\Delta t,x+\Delta r\right)dx-q\left(t,x\right)\right]dx & \approx \int_{r-\Delta r}^{r}[\frac{\partial q}{\partial t}\Delta t+\frac{\partial q}{\partial r}\Delta r]dx, \end{array}$$

assuming that the partial derivatives of *q* exist; they are evaluated at (*t*, *x*). From the mean-value theorem, we have for any integral

$$\begin{array}{c} \int_{x}^{x+\Delta x}f\left(z\right)dz=\Delta xf\left(\xi \right),\;x<\xi <x+\Delta x. \end{array}$$

Then, in our case, we get

$${\frac{\partial q}{\partial t}|}_{\left(t,\xi \right)}\Delta t\Delta r+{\frac{\partial q}{\partial r}|}_{\left(t,\xi \right)}\Delta r^{2}=-\rho \left(t,r\right)q\left(t,\xi \right)\Delta t\Delta r-\sqrt{\frac{\rho \left(t,r\right)q\left(t,\xi \right)\Delta r\Delta t}{N}}N\left(0,1\right),$$

where

$$\begin{array}{c} r-\Delta r<\xi <r. \end{array}$$

Note that

$$\begin{array}{c} \Delta r^{2}=\Delta t\Delta r \end{array}$$

because the refractory state increases linearly with *t* when neurons are not firing, hence

$$\begin{array}{c} \Delta r=\Delta t \end{array}$$

Dividing each side of this equation by Δ*t*Δ*r* and taking the limits

$$\begin{array}{c} \Delta t\to 0,\;\Delta r\to 0, \end{array}$$

yields the stochastic partial differential equation (SPDE) appearing as Eq 7 in the main text:

$$\frac{\partial q}{\partial t}+\frac{\partial q}{\partial r}=-\rho \left(t,r\right)q\left(t,r\right)-\sqrt{\frac{\rho (t,r)q(t,r)}{N}}\eta \left(t,r\right).$$

Here, we identified the limit of

$$\begin{array}{c} \eta \left(t,r\right)=\lim_{\Delta t\Delta r\to 0}\frac{1}{\sqrt{\Delta t\Delta r}}N\left(0,1\right), \end{array}$$

with a two-dimensional Gaussian white noise process *η*(*t*, *r*) obeying

$$\begin{array}{c} \left\langle \eta \left(t,r\right)\eta \left(t^{\prime },r^{\prime }\right)\right\rangle =\delta \left(t-t^{\prime }\right)\delta \left(r-r^{\prime }\right). \end{array}$$

The initial condition needed to solve the SPDE is obtained as follows. The total number of neurons firing in interval [*t*, *t* + Δ*t*), here denoted *n*(*t*), is given by

$$\begin{array}{c} n\left(t\right)=N\int_{t}^{t+\Delta t}A\left(t^{\prime }\right)dt^{\prime }, \end{array}$$ (24)

where *A*(*t*′) is the population activity. But *n*(*t*) is also the number of neurons at time *t* + Δ*t* with refractory state in [0, Δ*t*). Hence,

$$\begin{array}{c} m\left(t+\Delta t,\Delta t\right)=n\left(t\right)\Rightarrow N\int_{0}^{\Delta t}q\left(t+\Delta t,x\right)dx=N\int_{t}^{t+\Delta t}A\left(t^{\prime }\right)dt^{\prime }. \end{array}$$ (25)

In the limit of small Δ*t* we get

$$\begin{array}{c} q(t,0)=A(t), \end{array}$$ (26)

which is the required initial condition.

Moreover, *n*(*t*) must be equal to the summation of all neurons that fire in [*t*, *t* + Δ*t*), whatever their refractory state. Therefore, we must have, symbolically,

$$\begin{array}{c} N\int_{t}^{t+\Delta t}A\left(t^{\prime }\right)dt^{\prime }=\lim_{\Delta r\to 0}\sum_{r}\left[m\left(t,r\right)-m\left(t+\Delta t,r+\Delta r\right)\right]. \end{array}$$

Steps similar to those used above readily yield

$$\begin{array}{c} A\left(t\right)=\int_{0}^{\infty }dr\;[\rho \left(t,r\right)q\left(t,r\right)+\sqrt{\frac{\rho (t,r)q(t,r)}{N}}\eta \left(t,r\right)]. \end{array}$$ (27)

Finally, a normalization condition for *q*(*t*, *r*) is obtained from the fact that, at time *t*, all neurons within the network have a given refractory state:

$$\begin{array}{c} \int_{0}^{\infty }q\left(t,r\right)dr=1. \end{array}$$ (28)

Eqs 26–28—and variants thereof—are used in the main text.

Let us emphasize that we do not assume that the single neurons possess Poisson statistics. The Poisson random variable arises from an application of the theory of point processes. According to this theory, a generic point process with hazard function λ(t|H(t),Θ)—where H is the given history of events prior to *t* and Θ represents any covariate, e.g. an external stimulus—generates a spike in [*t*, *t* + Δ*t*) with probability $\lambda \left(t|H,\Theta \right)\Delta t+O\left(\Delta t^{2}\right)$ [46]. A Poisson process is obtained when the hazard function does not depend on history, in which case the hazard function identifies with the mean field itself (i.e. the firing rate). In our case, the hazard function *ρ* depends on the history

$$\begin{array}{c} H_{t}\equiv \left\{A\left(t^{\prime }\right):0<t^{\prime }<t\right\} \end{array}$$

and the external input through the potential *u*(*t*, *r*). Fundamentally, the number of neurons firing in [*t*, *t* + Δ*t*) follows a binomial distribution, which becomes a Poisson distribution in the limit Δ*t* → 0; the underlying Poisson random variable changes as a function of time and is correlated with other Poisson variables at other times (see appendix B in [35] for a mathematical explanation).

### Linear noise approximation

We start with the system-size expansion given in Eqs 11 and 12, that we rewrite here for convenience

$$q(t,r)=q_{0}\left(t,r\right)+\frac{1}{\sqrt{N}}q_{\xi }\left(t,r\right)+O(N^{-1})$$ (29)

$$A(t)=A_{0}\left(t\right)+\frac{1}{\sqrt{N}}A_{\xi }\left(t\right)+O(N^{-1}).$$ (30)

Replacing these two equations in the SPDE, Eq 7, yields

$$\begin{array}{c} \frac{dq_{0}}{dt}+\frac{1}{\sqrt{N}}\frac{dq_{\xi }}{dt}=-\rho (q_{0}+\frac{1}{\sqrt{N}}q_{\xi })-\sqrt{\frac{\rho (q_{0}+\frac{1}{\sqrt{N}}q_{\xi })}{N}}\eta \end{array}$$

omitting function arguments for brevity. Here, *d*/*dt* is the material derivative operator

$$\begin{array}{c} d/dt\equiv \partial /\partial t+\partial /\partial r. \end{array}$$

Using Eqs 30 and 4, we can write the hazard function as

$$\begin{array}{c} \begin{array}{cc} \rho [u(t,r)] & \approx \rho [\left(\kappa *\left[-J_{s}A_{0}+I_{\text{ext}}\right]\right)\left(t\right)-J_{s}N^{-1/2}\left(\kappa *A_{\xi }\right)\left(t\right)-V\left(r\right)]\\ & \approx \rho_{0}\left(t,r\right)-J_{s}N^{-1/2}\left(\kappa *A_{\xi }\right)\left(t\right)\frac{d\rho }{du}, \end{array} \end{array}$$ (31)

where

$$\begin{array}{c} \rho_{0}\left(t,r\right)\equiv \rho \left[u_{0}\left(t,r\right)\right] \end{array}$$

with

$$\begin{array}{c} u_{0}\left(t,r\right)\equiv \left(\kappa *\left[-J_{s}A_{0}+I_{\text{ext}}\right]\right)\left(t\right)-V\left(r\right) \end{array}$$

and the derivative *dρ*/*du* is evaluated at *u*_0_. Then, matching terms of orders O(1) and $O(N^{-1/2})$, and neglecting contributions from terms of order $O(N^{-1})$ and lower, we get two equations involving *q*_0_ and *q*_*ξ*_:

$$\begin{array}{c} \begin{array}{cc} \frac{dq_{0}}{dt} & =-\rho_{0}\left(t,r\right)q_{0}\left(t,r\right)\\ \frac{dq_{\xi }}{dt} & =-\rho_{0}\left(t,r\right)q_{\xi }\left(t,r\right)+J_{s}\left(\kappa *A_{\xi }\right)\left(t\right)\frac{d\rho }{du}q_{0}\left(t,r\right)-\sqrt{\rho_{0}\left(t,r\right)q_{0}\left(t,r\right)}\eta \left(t,r\right). \end{array} \end{array}$$ (32)

The first of these equations is the usual mean-field model (Eq 13). The second equation is a SPDE whose coefficients, source and noise terms depend on the solution of the deterministic equation. Of course, the boundary condition on *q*(*t*, *r*) (*cf.* Eqs 8 or 26) implies

$$\begin{array}{c} \begin{array}{cc} A_{0}\left(t\right) & =q_{0}\left(t,0\right)\\ A_{\xi }\left(t\right) & =q_{\xi }\left(t,0\right). \end{array} \end{array}$$ (33)

Furthermore, from Eq 9 and the LNA, we have, after algebraic manipulations:

$$\begin{array}{c} \begin{array}{cc} A_{0}\left(t\right) & =\int_{0}^{\infty }\rho_{0}\left(t,r\right)q_{0}\left(t,r\right)dr\\ A_{\xi }\left(t\right) & =\int_{0}^{\infty }\rho_{0}\left(t,r\right)q_{\xi }\left(t,r\right)-J_{s}\left(\kappa *A_{\xi }\right)\left(t\right)\int_{0}^{\infty }\frac{d\rho }{du}\left(t,r\right)q_{0}\left(t,r\right)dr\\ & \;+\int_{0}^{\infty }\sqrt{\rho_{0}\left(t,r\right)q_{0}\left(t,r\right)}\eta \left(t,r\right)dr. \end{array} \end{array}$$ (34)

Together, Eqs 32–34 constitute a coupled system between the deterministic mean field (*A*_0_ and *q*_0_) and the first-order fluctuations in the LNA (*A*_*ξ*_ and *q*_*ξ*_).

### Activity in the asynchronous state

Let us denote by *q*_∞_(*r*) and *A*_∞_ the refractory density and activity in the asynchronous steady state, respectively. We have to solve

$$\begin{array}{c} \frac{d}{dr}q_{\infty }\left(r\right)=-\rho_{\infty }\left(r\right)q_{\infty }\left(r\right), \end{array}$$ (35)

where

$$\begin{array}{c} \begin{array}{cc} \rho_{\infty }\left(r\right) & \equiv \rho \left[u_{\infty }\right]=\rho \left[-V\left(r\right)+h_{\infty }\right]\\ h_{\infty } & \equiv I_{\text{ext}}-J_{s}A_{\infty }. \end{array} \end{array}$$ (36)

Eq 35 can be integrated to give

$$\begin{array}{c} \begin{array}{c} q_{\infty }\left(r\right)=A_{\infty }e^{-\int_{0}^{r}\rho_{\infty }\left(s\right)\;ds}, \end{array} \end{array}$$ (37)

where we have used the boundary condition

$$\begin{array}{c} q_{\infty }\left(0\right)=A_{\infty }. \end{array}$$

Finally, the average asynchronous activity can be computed using the conservation property of the neural network, namely

$$\int_{0}^{\infty }q_{\infty }(r)dr=1,$$

so that

$$A_{\infty }^{-1}=\int_{0}^{\infty }e^{-\int_{0}^{r}\rho_{\infty }(s)\;ds}dr.$$

Note that the mean firing rate is implicitly given since *ρ*_∞_ depends on *A*_∞_. Therefore, to compute the mean activity, this nonlinear equation must be solved numerically.

With our choices for *ρ* we can push the calculation further. We have

$$\rho_{\infty }(r)=\lambda_{0}e^{h_{\infty }}\left(1-e^{-r/\tau }\right),$$

where *δu* has been set to its numerical value of 1 mV, hence

$$\int_{0}^{r}\rho_{\infty }(s)ds=\lambda_{0}e^{h_{\infty }}\left[r+\tau \left(e^{-r/\tau }-1\right)\right]$$

and

$$\begin{array}{ll} A_{\infty }^{-1} & =\int_{0}^{\infty }\text{exp}\left\{-\lambda_{0}e^{h_{\infty }}\left[r+\tau \left(e^{-r/\tau }-1\right)\right]\right\}dr\\ & =\text{exp}(\lambda_{0}e^{h_{\infty }}\tau )\int_{0}^{\infty }\text{exp}\left\{-\lambda_{0}e^{h_{\infty }}(r+\tau e^{-r/\tau })\right\}dr. \end{array}$$

The change of variable

$$t=\lambda_{0}\tau e^{h_{\infty }-r/\tau }$$

reduces the problem to finding the solution of the equation

$$A_{\infty }^{-1}=\tau {\left(\frac{e}{s_{\infty }}\right)}^{s_{\infty }}\gamma \left(s_{\infty },s_{\infty }\right),$$ (38)

where the lower incomplete γ function is given by

$$\gamma (a,x)=\int_{0}^{x}t^{a-1}e^{-t}dt,$$

and we defined

$$s_{\infty }\equiv \tau \rho_{\infty }(\infty )=\tau \lambda_{0}\text{exp}\left[I_{\text{ext}}-J_{s}A_{\infty }\right].$$

Eq 38 is then solved self-consistently for *A*_∞_.

### Stability analysis and the characteristic equation

To study the stability of the asynchronous state, one needs the eigenvalues of the differential operator once a linearization around the steady state has been performed. We therefore consider a small perturbation and write the solution in the form

$$\begin{array}{c} \begin{array}{c} q_{0}\left(t,r\right)=q_{\infty }\left(r\right)+\varepsilon q_{1}\left(t,r\right)+O\left(\varepsilon^{2}\right),\;A_{0}\left(t\right)=A_{\infty }+\varepsilon A_{1}\left(t\right)+O\left(\varepsilon^{2}\right). \end{array} \end{array}$$ (39)

Plugging these expressions into Eq 13—keeping the first order terms only—yields the partial differential equation

$$\begin{array}{c} \begin{array}{c} \frac{\partial }{\partial t}q_{1}\left(t,r\right)+\frac{\partial }{\partial r}q_{1}\left(t,r\right)=-\rho_{\infty }\left(r\right)q_{1}\left(t,r\right)+J_{s}{\frac{d\rho }{du}|}_{u_{\infty }\left(r\right)}q_{\infty }\left(r\right)\left(\kappa *A_{1}\right)\left(t\right), \end{array} \end{array}$$ (40)

with *ρ*_∞_(*r*) given by Eq 36 and

$$\begin{array}{c} u_{\infty }\left(r\right)=-V\left(r\right)+h_{\infty }. \end{array}$$

From Eq 40 (compare with Eqs 7 and 27), we have

$$\begin{array}{c} \begin{array}{c} A_{1}\left(t\right)=\int_{0}^{\infty }\rho_{\infty }\left(r\right)q_{1}\left(t,r\right)dr-J_{s}\left(\kappa *A_{1}\right)\left(t\right)\int_{0}^{\infty }{\frac{d\rho }{du}|}_{u_{\infty }\left(r\right)}q_{\infty }\left(r\right)dr. \end{array} \end{array}$$ (41)

We express the perturbation in eigenmodes

$$\begin{array}{c} \begin{array}{c} q_{1}\left(t,r\right)=e^{\lambda t}q_{1}\left(r\right),\;A_{1}\left(t\right)=e^{\lambda t}A_{1}. \end{array} \end{array}$$

After algebraic manipulations, we find that the perturbation obeys

$$\frac{d}{dr}q_{1}\left(r\right)=-\left[\rho_{\infty }\left(r\right)+\lambda \right]q_{1}\left(r\right)+J_{s}A_{1}{\frac{d\rho }{du}|}_{u_{\infty }\left(r\right)}q_{\infty }\left(r\right)\hat{\kappa }\left(\lambda \right),$$ (42)

with the Laplace transform of *κ* given by

$$\begin{array}{c} \hat{\kappa }\left(\lambda \right)\equiv \int_{0}^{\infty }\kappa \left(s\right)e^{-\lambda s}\;ds. \end{array}$$

Integration of this equation yields

$$q_{1}\left(r\right)=A_{1}S_{\infty }\left(r\right)e^{-\lambda r}+J_{s}A_{1}\hat{\kappa }\left(\lambda \right)\int_{0}^{r}{\frac{d\rho }{du}|}_{u_{\infty }\left(s\right)}q_{\infty }\left(s\right)e^{-\lambda \left(r-s\right)}\frac{S_{\infty }\left(r\right)}{S_{\infty }\left(s\right)}ds,$$ (43)

where

$$\begin{array}{c} S_{\infty }\left(r\right)\equiv \exp[-\int_{0}^{r}\rho_{\infty }\left(x\right)dx] \end{array}$$ (44)

is the survivor function in the steady state. From Eq 37, we have

$$\begin{array}{c} \begin{array}{c} q_{\infty }\left(s\right)=A_{\infty }S_{\infty }\left(s\right), \end{array} \end{array}$$

hence,

$$q_{1}\left(r\right)=A_{1}S_{\infty }\left(r\right)e^{-\lambda r}+J_{s}A_{1}\hat{\kappa }\left(\lambda \right)A_{\infty }S_{\infty }\left(r\right)\int_{0}^{r}{\frac{d\rho }{du}|}_{u_{\infty }\left(s\right)}e^{-\lambda \left(r-s\right)}ds.$$ (45)

Moreover, we get from Eq 41

$$\begin{array}{c} \begin{array}{c} A_{1}=\int_{0}^{\infty }\rho_{\infty }\left(r\right)q_{1}\left(r\right)dr-J_{s}\hat{\kappa }\left(\lambda \right)A_{1}\int_{0}^{\infty }{\frac{d\rho }{du}|}_{u_{\infty }\left(r\right)}q_{\infty }\left(r\right)dr. \end{array} \end{array}$$

Replacing Eq 45 into this latter equation gives, after cancellation of *A*_1_ throughout,

$$\begin{array}{ll} 1= & \int_{0}^{\infty }\rho_{\infty }\left(r\right)S_{\infty }\left(r\right)e^{-\lambda r}dr+J_{s}\hat{\kappa }\left(\lambda \right)A_{\infty }\int_{0}^{\infty }dr\;\rho_{\infty }\left(r\right)S_{\infty }\left(r\right)e^{-\lambda r}\int_{0}^{r}{\frac{d\rho }{du}|}_{u_{\infty }\left(s\right)}e^{\lambda s}ds\\ & -J_{s}\hat{\kappa }\left(\lambda \right)\int_{0}^{\infty }{\frac{d\rho }{du}|}_{u_{\infty }\left(r\right)}q_{\infty }\left(r\right)dr. \end{array}$$

Writing

$$\begin{array}{c} P_{\infty }\left(r\right)\equiv \rho_{\infty }\left(r\right)S_{\infty }\left(r\right) \end{array}$$ (46)

for the probability density of obtaining a refractory state *r* in the steady state and defining its Laplace transform

$$\begin{array}{c} {\hat{P}}_{\infty }\left(\lambda \right)\equiv \int_{0}^{\infty }P_{\infty }\left(r\right)e^{-\lambda r}dr, \end{array}$$ (47)

we get

$$\begin{array}{ll} 1= & {\hat{P}}_{\infty }\left(\lambda \right)+J_{s}\hat{\kappa }\left(\lambda \right)A_{\infty }\int_{0}^{\infty }dr\;P_{\infty }\left(r\right)e^{-\lambda r}\int_{0}^{r}ds{\frac{d\rho }{du}|}_{u_{\infty }\left(s\right)}e^{\lambda s}\\ & -J_{s}\hat{\kappa }\left(\lambda \right)\int_{0}^{\infty }{\frac{d\rho }{du}|}_{u_{\infty }\left(r\right)}q_{\infty }\left(r\right)dr. \end{array}$$

We shall write the characteristic equation as C(λ)=0 with

$$\begin{array}{ll} C\left(\lambda \right) & \equiv 1-{\hat{P}}_{\infty }\left(\lambda \right)-J_{s}\hat{\kappa }\left(\lambda \right)A_{\infty }\int_{0}^{\infty }dr\int_{0}^{r}ds\;P_{\infty }\left(r\right){\frac{d\rho }{du}|}_{u_{\infty }\left(s\right)}e^{-\lambda \left(r-s\right)}\\ & +J_{s}\hat{\kappa }\left(\lambda \right)\int_{0}^{\infty }{\frac{d\rho }{du}|}_{u_{\infty }\left(r\right)}q_{\infty }\left(r\right)dr. \end{array}$$

The eigenvalues are thus given by the roots of C(λ). The time-independent solution *A*_∞_ will be stable if all the eigenvalues have negative real parts.

### Computation of the power spectrum

We assume that the deterministic part of the activity tends toward its equilibrium state—below the instability threshold. In other words, *t* is large enough that

$$\begin{array}{c} q_{0}\left(t,r\right)=q_{\infty }\left(r\right),\;A_{0}\left(t\right)=A_{\infty }. \end{array}$$

Thus discarding the transient dynamics, the SPDE involving the finite-size fluctuations in the LNA (see second equation of Eq 32) becomes

$$\frac{\partial q_{\xi }}{\partial t}+\frac{\partial q_{\xi }}{\partial r}=-\rho_{\infty }\left(r\right)q_{\xi }\left(t,r\right)+J_{s}\left(\kappa *A_{\xi }\right)\left(t\right){\frac{d\rho }{du}|}_{u_{\infty }}q_{\infty }\left(r\right)-\sqrt{\rho_{\infty }\left(r\right)q_{\infty }\left(r\right)}\eta \left(t,r\right).$$ (48)

On the other hand, the stochastic part of the activity now reads (see second equation in Eq 34)

$$\begin{array}{ll} A_{\xi }\left(t\right) & {=\int }_{0}^{\infty }\rho_{\infty }\left(r\right)q_{\xi }\left(t,r\right)dr-J_{s}\left(\kappa *A_{\xi }\right)\left(t\right)\int_{0}^{\infty }{\frac{d\rho }{du}|}_{u_{\infty }\left(r\right)}q_{\infty }\left(r\right)dr\\ & +\int_{0}^{\infty }\sqrt{\rho_{\infty }\left(r\right)q_{\infty }\left(r\right)}\eta \left(t,r\right)dr. \end{array}$$ (49)

The power spectrum is defined by

$$P_{\xi }\left(\omega \right)\equiv \underset{T\to \infty }{\text{lim}}\frac{\langle {\left|{\tilde{A}}_{\xi }\left(\omega \right)\right|}^{2}\rangle }{T},$$

where

$$\begin{array}{c} {\tilde{A}}_{\xi_{T}}\left(\omega \right)\equiv \int_{0}^{T}A_{\xi }\left(t\right)e^{-i\omega t}dt \end{array}$$ (50)

is the Fourier transform of the stochastic process *A*_*ξ*_ restricted to 0 < *t* < *T*. Analogously, we define

$$\begin{array}{c} {\tilde{q}}_{\xi_{T}}\left(\omega ,r\right)\equiv \int_{0}^{T}q_{\xi }\left(t,r\right)e^{-i\omega t}\;dt, \end{array}$$

and likewise for other stochastic processes depending on *t* and *r*. After taking the Fourier transform, Eq 48 becomes (arguments of functions are omitted when no confusion may arise)

$$\frac{\partial {\tilde{q}}_{\xi_{T}}}{\partial r}=-\left[\rho_{\infty }\left(r\right)+i\omega \right]{\tilde{q}}_{\xi_{T}}+J_{s}\tilde{\kappa }{\tilde{A}}_{\xi_{T}}{\frac{d\rho }{du}|}_{u_{\infty }}q_{\infty }\left(r\right)-\sqrt{\rho_{\infty }\left(r\right)q_{\infty }\left(r\right)}{\tilde{\eta }}_{T}\left(\omega ,r\right).$$

Note that this equation has exactly the same form as Eq 42 above, and thus can be solved accordingly:

$$\begin{array}{ll} {\tilde{q}}_{\xi_{T}}= & {\tilde{A}}_{\xi_{T}}S_{\infty }\left(r\right)e^{-i\omega r}+J_{s}\tilde{\kappa }{\tilde{A}}_{\xi_{T}}A_{\infty }\int_{0}^{r}{\frac{d\rho }{du}|}_{u_{\infty }\left(s\right)}e^{-i\omega \left(r-s\right)}S_{\infty }\left(r\right)ds\\ & -\int_{0}^{r}\sqrt{\rho_{\infty }\left(s\right)q_{\infty }\left(s\right)}{\tilde{\eta }}_{T}\left(\omega ,s\right)e^{-i\omega \left(r-s\right)}\frac{S_{\infty }\left(r\right)}{S_{\infty }\left(s\right)}ds, \end{array}$$

where we used

$$\begin{array}{c} {\tilde{q}}_{\xi_{T}}\left(\omega ,0\right)={\tilde{A}}_{\xi_{T}}\left(\omega \right). \end{array}$$

Also, from Eq 49 we get

$$\begin{array}{ll} {\tilde{A}}_{\xi_{T}}= & \int_{0}^{\infty }\rho_{\infty }\left(r\right){\tilde{q}}_{\xi_{T}}\left(\omega ,r\right)dr-J_{s}\tilde{\kappa }{\tilde{A}}_{\xi_{T}}\int_{0}^{\infty }{\frac{d\rho }{du}|}_{u_{\infty }\left(r\right)}q_{\infty }\left(r\right)dr\\ & +\int_{0}^{\infty }\sqrt{\rho_{\infty }\left(r\right)q_{\infty }\left(r\right)}{\tilde{\eta }}_{T}\left(\omega ,r\right)dr. \end{array}$$ (51)

Replacing ${\tilde{q}}_{\xi_{T}}$ in this equation by the expression that has just been obtained for it yields

$$\begin{array}{ll} {\tilde{A}}_{\xi_{T}} & ={\tilde{A}}_{\xi_{T}}\int_{0}^{\infty }P_{\infty }\left(r\right)e^{-i\omega r}dr+J_{s}\tilde{\kappa }{\tilde{A}}_{\xi_{T}}A_{\infty }\int_{0}^{\infty }\int_{0}^{r}{\frac{d\rho }{du}|}_{u_{\infty }\left(s\right)}e^{-i\omega \left(r-s\right)}P_{\infty }\left(r\right)dsdr\\ & -\int_{0}^{\infty }\int_{0}^{r}\sqrt{\rho_{\infty }\left(s\right)q_{\infty }\left(s\right)}{\tilde{\eta }}_{T}\left(\omega ,s\right)e^{-i\omega \left(r-s\right)}\frac{P_{\infty }\left(r\right)}{S_{\infty }\left(s\right)}dsdr-J_{s}\tilde{\kappa }{\tilde{A}}_{\xi_{T}}\int_{0}^{\infty }{\frac{d\rho }{du}|}_{u_{\infty }\left(r\right)}q_{\infty }\left(r\right)dr\\ & +\int_{0}^{\infty }\sqrt{\rho_{\infty }\left(r\right)q_{\infty }\left(r\right)}{\tilde{\eta }}_{T}\left(\omega ,r\right)dr. \end{array}$$

Hence, with

$$\begin{array}{c} {\tilde{P}}_{\infty }\left(\omega \right)={\hat{P}}_{\infty }\left(i\omega \right)=\int_{0}^{\infty }P_{\infty }\left(r\right)e^{-i\omega r}dr \end{array}$$

the characteristic function of *P*_∞_(*r*), we get

$$\begin{array}{c} {\tilde{A}}_{\xi_{T}}\left(\omega \right)=\frac{\int_{0}^{\infty }\sqrt{\rho_{\infty }\left(r\right)q_{\infty }\left(r\right)}{\tilde{\eta }}_{T}\left(\omega ,r\right)dr-\int_{0}^{\infty }\int_{0}^{r}\sqrt{\rho_{\infty }\left(s\right)q_{\infty }\left(s\right)}{\tilde{\eta }}_{T}\left(\omega ,s\right)e^{-i\omega (r-s)}\frac{P_{\infty }\left(r\right)}{S_{\infty }\left(s\right)}dsdr}{C(i\omega )}. \end{array}$$

To simplify the notation, we define

$$\begin{array}{c} \alpha_{\omega }\left(s,r\right)\equiv e^{-i\omega (r-s)}\frac{P_{\infty }\left(r\right)}{S_{\infty }\left(s\right)},\;f\left(r\right)\equiv \sqrt{\rho_{\infty }\left(r\right)q_{\infty }\left(r\right)}. \end{array}$$

Then,

$$\begin{array}{c} {\tilde{A}}_{\xi_{T}}\left(\omega \right)=\frac{\int_{0}^{\infty }f\left(r\right){\tilde{\eta }}_{T}\left(\omega ,r\right)dr-\int_{0}^{\infty }\int_{0}^{r}f\left(s\right){\tilde{\eta }}_{T}\left(\omega ,s\right)\alpha_{\omega }\left(s,r\right)dsdr}{C(i\omega )}. \end{array}$$

To compute the power spectrum of *A*_*ξ*_, we first note that

$$\begin{array}{c} \lim_{T\to \infty }\frac{\left\langle {\tilde{\eta }}_{T}\left(\omega ,r\right){\tilde{\eta }}_{T}^{*}\left(\omega ,r^{\prime }\right)\right\rangle }{T}=\delta \left(r-r^{\prime }\right) \end{array}$$

since *η*(*t*, *r*) is a Gaussian white noise. Also, in the expression for ${\tilde{A}}_{\xi_{T}}\left(\omega \right)$, only the numerator contains stochastic terms. Therefore, we only have to compute

$$\underset{T\to \infty }{\text{lim}}\frac{1}{T}\langle {\left|\int_{0}^{\infty }f\left(r\right){\tilde{\eta }}_{T}\left(\omega ,r\right)dr-\int_{0}^{\infty }\int_{0}^{r}f\left(s\right){\tilde{\eta }}_{T}\left(\omega ,s\right)\alpha_{\omega }\left(s,r\right)dsdr\right|}^{2}\rangle$$

which gives rise to four terms that we compute separately (we denote $\langle \cdot \rangle \equiv \lim_{T\to \infty }\frac{1}{T}\langle \cdot \rangle$).

**First term**

$$\begin{array}{c} \int_{0}^{\infty }\int_{0}^{\infty }dr\;dr^{\prime }\;f\left(r\right)f\left(r^{\prime }\right)\langle {\tilde{\eta }}_{T}\left(\omega ,r\right){\tilde{\eta }}_{T}^{*}\left(\omega ,r^{\prime }\right)\rangle \\ =\int_{0}^{\infty }\int_{0}^{\infty }dr\;dr^{\prime }\;f\left(r\right)f\left(r^{\prime }\right)\delta \left(r-r^{\prime }\right)\\ =\int_{0}^{\infty }{\left[f\left(r\right)\right]}^{2}dr=A_{\infty }. \end{array}$$

**Second term**

$$\begin{array}{c} \int_{0}^{\infty }\int_{0}^{\infty }\int_{0}^{r^{\prime }}drdr^{\prime }ds^{\prime }f\left(r\right)f\left(s^{\prime }\right)\alpha_{\omega }^{*}\left(s^{\prime },r^{\prime }\right)\delta \left(r-s^{\prime }\right)\\ =\int_{0}^{\infty }\int_{0}^{\infty }drdr^{\prime }{\left[f\left(r\right)\right]}^{2}\alpha_{\omega }^{*}\left(r,r^{\prime }\right)H\left(r^{\prime }-r\right)\\ =\int_{0}^{\infty }dr\int_{0}^{r}dr^{\prime }{\left[f\left(r\right)\right]}^{2}\alpha_{\omega }^{*}\left(r,r^{\prime }\right) \end{array}$$

since

$$\begin{array}{c} \int_{0}^{r^{\prime }}ds^{\prime }g\left(s^{\prime }\right)\delta \left(r-s^{\prime }\right)=g\left(r\right)H\left(r^{\prime }-r\right) \end{array}$$

for an arbitrary function *g*(*s*).

**Third term**

$$\begin{array}{c} \int_{0}^{\infty }\int_{0}^{\infty }\int_{0}^{r^{\prime }}drdr^{\prime }ds^{\prime }f\left(r\right)f\left(s^{\prime }\right)\alpha_{\omega }\left(s^{\prime },r^{\prime }\right)\delta \left(r-s^{\prime }\right)\\ =\int_{0}^{\infty }dr\int_{0}^{r}dr^{\prime }{\left[f\left(r\right)\right]}^{2}\alpha_{\omega }\left(r,r^{\prime }\right). \end{array}$$

**Fourth term**

$$\begin{array}{l} \int_{0}^{\infty }dr\int_{0}^{\infty }dr^{\prime }\int_{0}^{r^{\prime }}ds^{\prime }\int_{0}^{r}dsf\left(s\right)f\left(s^{\prime }\right)\alpha_{\omega }\left(s,r\right)\alpha_{\omega }^{*}\left(s^{\prime },r^{\prime }\right)\delta \left(s-s^{\prime }\right)\\ =\int_{0}^{\infty }dr\int_{0}^{\infty }dr^{\prime }\int_{0}^{r^{\prime }}ds{\left[f\left(s\right)\right]}^{2}\alpha_{\omega }\left(s,r\right)\alpha_{\omega }^{*}\left(s,r^{\prime }\right)H\left(r-s\right)\\ =\int_{0}^{\infty }ds\int_{s}^{\infty }dr{\left[f\left(s\right)\right]}^{2}\alpha_{\omega }\left(s,r\right)\int_{s}^{\infty }dr^{\prime }\alpha_{\omega }^{*}\left(s,r^{\prime }\right)\\ =\int_{0}^{\infty }ds{\left[f\left(s\right)\right]}^{2}{\left|\int_{s}^{\infty }dr\alpha_{\omega }\left(s,r\right)\right|}^{2}. \end{array}$$

The power spectrum is then

$$P_{\xi }\left(\omega \right)=\frac{A_{\infty }+\int_{0}^{\infty }ds\;{\left[f\left(s\right)\right]}^{2}{\left|\int_{s}^{\infty }dr\alpha_{\omega }\left(s,r\right)\right|}^{2}-2ℜ\int_{0}^{\infty }ds\;{\left[f\left(s\right)\right]}^{2}\int_{0}^{s}dr\alpha_{\omega }\left(s,r\right)}{{\left|C\left(i\omega \right)\right|}^{2}}.$$

With the definitions for *f*(*s*) and *α*_*ω*_(*s*, *r*), the second term of the numerator becomes

$$\begin{array}{l} \int_{0}^{\infty }ds\;\rho_{\infty }\left(s\right)q_{\infty }\left(s\right){\left|\int_{s}^{\infty }dr\;e^{-i\omega \left(r-s\right)}\frac{P_{\infty }\left(r\right)}{S_{\infty }\left(s\right)}\right|}^{2}\\ =A_{\infty }\int_{0}^{\infty }ds\;\frac{\rho_{\infty }\left(s\right)}{S_{\infty }\left(s\right)}{\left|\int_{s}^{\infty }dr\;e^{-i\omega r}P_{\infty }\left(r\right)\right|}^{2} \end{array}$$

and for the third term,

$$\begin{array}{c} 2ℜ\{A_{\infty }\int_{0}^{\infty }ds\;\rho_{\infty }\left(s\right)e^{i\omega s}\int_{0}^{s}dr\;e^{-i\omega r}P_{\infty }\left(r\right)\}. \end{array}$$

Hence, finally,

$$\begin{array}{ll} P_{\xi }\left(\omega \right)= & A_{\infty }\frac{1+\int_{0}^{\infty }ds\;\rho_{\infty }\left(s\right)e^{\int_{0}^{s}\rho_{\infty }\left(x\right)dx}{\left|\int_{s}^{\infty }dr\;e^{-i\omega r}P_{\infty }\left(r\right)\right|}^{2}}{{\left|C\left(i\omega \right)\right|}^{2}}\\ & -A_{\infty }\frac{2ℜ\left\{\int_{0}^{\infty }ds\int_{0}^{s}dr\;\rho_{\infty }\left(s\right)P_{\infty }\left(r\right)e^{-i\omega \left(r-s\right)}\right\}}{{\left|C\left(i\omega \right)\right|}^{2}}. \end{array}$$

### Autocorrelation function

In this section, we compute *A*_*ξ*_ and (a part of) its autocorrelation function. According to the Wiener-Khintchin theorem, the autocorrelation function of *A*_*ξ*_ is simply the Fourier transform of its power spectrum. But to allow comparison with the results in [35], we calculate a part of this autocorrelation directly. From Eq 49, we must determine *q*_*ξ*_(*t*, *r*). This will be done by first solving Eq 48 using the method of characteristics.

#### Solving the first-order inhomogeneous PDE with the method of characteristics

We have to solve

$$\begin{array}{c} \begin{array}{cc} \frac{\partial q_{\xi }}{\partial t}+\frac{\partial q_{\xi }}{\partial r} & =-\rho_{\infty }\left(r\right)q_{\xi }\left(t,r\right)+F\left(t,r\right). \end{array} \end{array}$$ (52)

where

$$\begin{array}{c} F\left(t,r\right)\equiv J_{s}\left(\kappa *A_{\xi }\right)\left(t\right){\frac{d\rho }{du}|}_{u_{\infty }\left(r\right)}q_{\infty }\left(r\right)-\sqrt{\rho_{\infty }\left(r\right)q_{\infty }\left(r\right)}\eta \left(t,r\right). \end{array}$$

The ranges of *r* and *t* are [0, ∞) and (−∞, ∞), respectively. The boundary condition is

$$\begin{array}{c} q_{\xi }\left(t,0\right)=A_{\xi }\left(t\right). \end{array}$$

With the method of characteristics, we first transform the PDE above into an ordinary differential equation. We find a family of curves for which

$$\begin{array}{c} \frac{\partial q_{\xi }}{\partial t}+\frac{\partial q_{\xi }}{\partial r}=\frac{dq_{\xi }}{dr}. \end{array}$$

Since

$$\begin{array}{c} dt/dr=1, \end{array}$$

we have

$$\begin{array}{c} t=r+t_{0}. \end{array}$$

Along these lines,

$$\begin{array}{c} \frac{d}{dr}q_{\xi }\left(t\left(r\right),r\right)=\frac{\partial q_{\xi }}{\partial t}\frac{dt}{dr}+\frac{\partial q_{\xi }}{\partial r}. \end{array}$$

Hence, we now have to solve the ODE on the characteristic curve, namely

$$\begin{array}{c} \frac{dq_{\xi }}{dr}+\rho_{\infty }\left(r\right)q_{\xi }=F\left(r+t_{0},r\right),\;q_{\xi }\left(t_{0},0\right)=A_{\xi }\left(t_{0}\right). \end{array}$$

The solution is

$$\begin{array}{c} q_{\xi }\left(t\left(r\right),r\right)=A_{\xi }\left(t_{0}\right)S_{\infty }\left(r\right)+S_{\infty }\left(r\right)\int_{0}^{r}\frac{F(\tau +t_{0},\tau )}{S_{\infty }\left(\tau \right)}d\tau . \end{array}$$

Replacing

$$\begin{array}{c} t_{0}=t-r, \end{array}$$

we get the solution for the whole (*t*, *r*) space:

$$\begin{array}{c} q_{\xi }\left(t,r\right)=A_{\xi }\left(t-r\right)S_{\infty }\left(r\right)+S_{\infty }\left(r\right)\int_{0}^{r}\frac{F(\tau +t-r,\tau )}{S_{\infty }\left(\tau \right)}d\tau . \end{array}$$

Replacing this expression into Eq 49 yields

$$\begin{array}{ll} A_{\xi }\left(t\right)= & \int_{0}^{\infty }A_{\xi }\left(t-r\right)P_{\infty }\left(r\right)dr+\int_{0}^{\infty }P_{\infty }\left(r\right)\int_{0}^{r}\frac{F\left(\tau +t-r,\tau \right)}{S_{\infty }\left(\tau \right)}d\tau dr\\ & -J_{s}\left(\kappa *A_{\xi }\right)\left(t\right)\int_{0}^{\infty }{\frac{d\rho }{du}|}_{u_{\infty }\left(r\right)}q_{\infty }\left(r\right)dr+\int_{0}^{\infty }\sqrt{\rho_{\infty }\left(r\right)q_{\infty }\left(r\right)}\eta \left(t,r\right)dr. \end{array}$$ (53)

The second term is

$$\begin{array}{l} \int_{0}^{\infty }P_{\infty }\left(r\right)\int_{0}^{r}\frac{F\left(\tau +t-r,\tau \right)}{S_{\infty }\left(\tau \right)}d\tau dr\\ =\int_{0}^{\infty }P_{\infty }\left(r\right)\int_{0}^{r}\frac{J_{s}\left(\kappa *A_{\xi }\right)\left(\tau +t-r\right){\frac{d\rho }{du}|}_{u_{\infty }\left(\tau \right)}q_{\infty }\left(\tau \right)-\sqrt{\rho_{\infty }\left(\tau \right)q_{\infty }\left(\tau \right)}\eta \left(\tau +t-r,\tau \right)}{S_{\infty }\left(\tau \right)}d\tau dr. \end{array}$$

The first part of this second term can be written

$$\begin{array}{l} J_{s}A_{\infty }\int_{0}^{\infty }dr\;P_{\infty }\left(r\right)\int_{0}^{r}d\tau \;\int_{-\infty }^{\infty }dx\;\kappa \left(\tau +t-r-x\right)A_{\xi }\left(x\right)\frac{d\rho }{du}{|}_{u_{\infty }\left(\tau \right)}\\ \equiv J_{s}A_{\infty }\left(G*A_{\xi }\right)\left(t\right) \end{array}$$

with

$$G\left(t\right)\equiv \int_{0}^{\infty }drP_{\infty }\left(r\right)\int_{0}^{r}d\tau {\frac{d\rho }{du}|}_{u_{\infty }\left(\tau \right)}\kappa \left(t+\tau -r\right).$$

Therefore, we finally have

$$\begin{array}{c} A_{\xi }\left(t\right)=\left(Q*A_{\xi }\right)\left(t\right)+\Sigma \left(t\right) \end{array}$$ (54)

with

$$\begin{array}{ll} Q\left(t\right) & \equiv P_{\infty }\left(t\right)-J_{s}\left[a_{\infty }\kappa \left(t\right)-A_{\infty }G\left(t\right)\right],\\ a_{\infty } & \equiv \int_{0}^{\infty }{\frac{d\rho }{du}|}_{u_{\infty }\left(r\right)}q_{\infty }\left(r\right)dr, \end{array}$$

and

$$\begin{array}{c} \Sigma \left(t\right)\equiv \int_{0}^{\infty }\sqrt{\rho_{\infty }\left(r\right)q_{\infty }\left(r\right)}\eta \left(t,r\right)dr-\int_{0}^{\infty }dr\;P_{\infty }\left(r\right)\int_{0}^{r}d\tau \frac{\sqrt{\rho_{\infty }\left(\tau \right)q_{\infty }\left(\tau \right)}\eta \left(\tau +t-r,\tau \right)}{S_{\infty }\left(\tau \right)}. \end{array}$$

In the case where the hazard function is exponential, as in Eq 5, *a*_∞_ becomes

$$\begin{array}{c} a_{\infty }=\frac{1}{\delta u}\int_{0}^{\infty }\rho_{\infty }\left(r\right)q_{\infty }\left(r\right)dr=\frac{A_{\infty }}{\delta u} \end{array}$$

whereas

$$\begin{array}{c} G\left(t\right)=\frac{1}{\delta u}\int_{0}^{\infty }drP_{\infty }\left(r\right)\int_{0}^{r}d\tau \rho_{\infty }\left(\tau \right)\kappa \left(t+\tau -r\right). \end{array}$$

#### Autocorrelation function of Σ

We compute 〈Σ(*t*)Σ(*t* + *t*′)〉:

$$\begin{array}{l} \langle \Sigma \left(t\right)\Sigma \left(t+t^{\prime }\right)\rangle =\\ \langle \left[\int_{0}^{\infty }\sqrt{\rho_{\infty }\left(r\right)q_{\infty }\left(r\right)}\eta \left(t,r\right)dr-\int_{0}^{\infty }P_{\infty }\left(r\right)\int_{0}^{r}\frac{\sqrt{\rho_{\infty }\left(\tau \right)q_{\infty }\left(\tau \right)}\eta \left(\tau +t-r,\tau \right)}{S_{\infty }\left(\tau \right)}d\tau dr\right]\\ \left[\int_{0}^{\infty }\sqrt{\rho_{\infty }\left(r^{\prime }\right)q_{\infty }\left(r^{\prime }\right)}\eta \left(t+t^{\prime },r^{\prime }\right)dr^{\prime }-\int_{0}^{\infty }P_{\infty }\left(r^{\prime }\right)\int_{0}^{r^{\prime }}\frac{\sqrt{\rho_{\infty }\left(\tau^{\prime }\right)q_{\infty }\left(\tau^{\prime }\right)}\eta \left(\tau^{\prime }+t+t^{\prime }-r^{\prime },\tau^{\prime }\right)}{S_{\infty }\left(\tau^{\prime }\right)}d\tau^{\prime }dr^{\prime }\right]\rangle \end{array}$$

First, we have

$$\begin{array}{c} \int_{0}^{\infty }\int_{0}^{\infty }\sqrt{\rho_{\infty }\left(r\right)q_{\infty }\left(r\right)}\sqrt{\rho_{\infty }\left(r^{\prime }\right)q_{\infty }\left(r^{\prime }\right)}\left\langle \eta \left(t,r\right)\eta \left(t+t^{\prime },r^{\prime }\right)\right\rangle dr^{\prime }dr\\ =\int_{0}^{\infty }\int_{0}^{\infty }\sqrt{\rho_{\infty }\left(r\right)q_{\infty }\left(r\right)}\sqrt{\rho_{\infty }\left(r^{\prime }\right)q_{\infty }\left(r^{\prime }\right)}\delta \left(t^{\prime }\right)\delta \left(r-r^{\prime }\right)dr^{\prime }dr\\ =\delta \left(t^{\prime }\right)\int_{0}^{\infty }\rho_{\infty }\left(r\right)q_{\infty }\left(r\right)dr=A_{\infty }\delta \left(t^{\prime }\right). \end{array}$$

Second, we have

$$\begin{array}{c} -\int_{0}^{\infty }\int_{0}^{\infty }\int_{0}^{r^{\prime }}\sqrt{\rho_{\infty }\left(r\right)q_{\infty }\left(r\right)}P_{\infty }\left(r^{\prime }\right)\frac{\sqrt{\rho_{\infty }\left(\tau^{\prime }\right)q_{\infty }\left(\tau^{\prime }\right)}}{S_{\infty }\left(\tau^{\prime }\right)}\left\langle \eta \left(t,r\right)\eta \left(\tau^{\prime }+t+t^{\prime }-r^{\prime },\tau^{\prime }\right)\right\rangle d\tau^{\prime }dr^{\prime }dr\\ =-\int_{0}^{\infty }\int_{0}^{\infty }\int_{0}^{r^{\prime }}\sqrt{\rho_{\infty }\left(r\right)q_{\infty }\left(r\right)}P_{\infty }\left(r^{\prime }\right)\frac{\sqrt{\rho_{\infty }\left(\tau^{\prime }\right)q_{\infty }\left(\tau^{\prime }\right)}}{S_{\infty }\left(\tau^{\prime }\right)}\delta \left(\tau^{\prime }+t^{\prime }-r^{\prime }\right)\delta \left(r-\tau^{\prime }\right)d\tau^{\prime }dr^{\prime }dr\\ =-A_{\infty }\int_{0}^{\infty }\int_{0}^{r^{\prime }}P_{\infty }\left(r^{\prime }\right)\rho_{\infty }\left(\tau^{\prime }\right)\delta \left(\tau^{\prime }+t^{\prime }-r^{\prime }\right)d\tau^{\prime }dr^{\prime }\\ =-A_{\infty }H\left(t^{\prime }\right)\int_{0}^{\infty }H\left(r-t^{\prime }\right)P_{\infty }\left(r\right)\rho_{\infty }\left(r-t^{\prime }\right)dr \end{array}$$

Similarly, for the third term we obtain

$$\begin{array}{c} -A_{\infty }H\left(-t^{\prime }\right)\int_{0}^{\infty }H\left(r+t^{\prime }\right)P_{\infty }\left(r\right)\rho_{\infty }\left(r+t^{\prime }\right)dr. \end{array}$$

We can combine the last two expressions by writing

$$\begin{array}{c} -A_{\infty }\int_{0}^{\infty }H(r-|t^{\prime }\left|\right)P_{\infty }\left(r\right)\rho_{\infty }\left(r-\right|t^{\prime }\left|\right)dr\\ =-A_{\infty }\int_{0}^{\infty }\rho_{\infty }\left(r\right)P_{\infty }\left(r+\right|t^{\prime }\left|\right)dr. \end{array}$$

For the fourth term, defining

$$\begin{array}{c} \sqrt{\rho_{\infty }\left(r\right)q_{\infty }\left(r\right)}\equiv s\left(r\right), \end{array}$$

we have

$$\begin{array}{c} \int_{0}^{\infty }\int_{0}^{\infty }\int_{0}^{r}\int_{0}^{r^{\prime }}P_{\infty }\left(r\right)P_{\infty }\left(r^{\prime }\right)\frac{s(\tau )}{S_{\infty }\left(\tau \right)}\frac{s(\tau^{\prime })}{S_{\infty }\left(\tau^{\prime }\right)}\left\langle \eta \left(\tau +t-r,\tau \right)\eta \left(\tau^{\prime }+t+t^{\prime }-r^{\prime },\tau^{\prime }\right)\right\rangle d\tau^{\prime }d\tau drdr^{\prime }\\ =\int_{0}^{\infty }\int_{0}^{\infty }\int_{0}^{r}\int_{0}^{r^{\prime }}P_{\infty }\left(r\right)P_{\infty }\left(r^{\prime }\right)\frac{s(\tau )}{S_{\infty }\left(\tau \right)}\frac{s(\tau^{\prime })}{S_{\infty }\left(\tau^{\prime }\right)}\delta \left(\tau^{\prime }+t^{\prime }-r^{\prime }-\tau +r\right)\delta \left(\tau -\tau^{\prime }\right)d\tau^{\prime }d\tau drdr^{\prime }\\ =\int_{0}^{\infty }\int_{0}^{\infty }\int_{0}^{r}H\left(r^{\prime }-\tau \right)P_{\infty }\left(r\right)P_{\infty }\left(r^{\prime }\right)\frac{s{\left(\tau \right)}^{2}}{S_{\infty }{\left(\tau \right)}^{2}}\delta \left(t^{\prime }+r-r^{\prime }\right)d\tau drdr^{\prime }\\ =A_{\infty }\int_{0}^{\infty }dr\int_{0}^{r}d\tau \;H\left(r+t^{\prime }-\tau \right)P_{\infty }\left(r\right)P_{\infty }\left(r+t^{\prime }\right)\frac{\rho_{\infty }\left(\tau \right)}{S_{\infty }\left(\tau \right)}, \end{array}$$

where in the last line we used

$$\begin{array}{c} q_{\infty }\left(r\right)=S_{\infty }\left(r\right)A_{\infty }. \end{array}$$

When *t*′ > 0, we have

$$\begin{array}{c} A_{\infty }\int_{0}^{\infty }dr\;P_{\infty }\left(r\right)P_{\infty }\left(r+t^{\prime }\right)\int_{0}^{r}d\tau \;\frac{\rho_{\infty }\left(\tau \right)}{S_{\infty }\left(\tau \right)}\\ =A_{\infty }\int_{0}^{\infty }dr\;\rho_{\infty }\left(r\right)P_{\infty }\left(r+t^{\prime }\right)-A_{\infty }\int_{0}^{\infty }dr\;P_{\infty }\left(r\right)P_{\infty }\left(r+t^{\prime }\right) \end{array}$$

where we used

$$\begin{array}{c} \int_{0}^{r}d\tau \;\frac{\rho_{\infty }\left(\tau \right)}{S_{\infty }\left(\tau \right)}=-1+1/S_{\infty }\left(r\right). \end{array}$$

When *t*′ < 0,

$$\begin{array}{c} A_{\infty }\int_{0}^{\infty }dr\;P_{\infty }\left(r\right)P_{\infty }\left(r-\right|t^{\prime }\left|\right)\int_{0}^{r-|t^{\prime }|}d\tau \;\frac{\rho_{\infty }\left(\tau \right)}{S_{\infty }\left(\tau \right)}\\ =A_{\infty }\int_{0}^{\infty }dr\;\rho_{\infty }\left(r-\right|t^{\prime }\left|\right)P_{\infty }\left(r\right)-A_{\infty }\int_{0}^{\infty }dr\;P_{\infty }\left(r\right)P_{\infty }\left(r-\right|t^{\prime }\left|\right)\\ =A_{\infty }\int_{0}^{\infty }dr\;\rho_{\infty }\left(r\right)P_{\infty }\left(r+\right|t^{\prime }\left|\right)-A_{\infty }\int_{0}^{\infty }dr\;P_{\infty }\left(r\right)P_{\infty }\left(r+\right|t^{\prime }\left|\right). \end{array}$$

The autocorrelation function is then

$$\begin{array}{ll} \langle \Sigma \left(t\right)\Sigma \left(t+t^{\prime }\right)\rangle & =A_{\infty }\delta \left(t^{\prime }\right)-A_{\infty }\int_{0}^{\infty }\rho_{\infty }\left(r\right)P_{\infty }(r+|t^{\prime }|)dr\\ & +A_{\infty }\int_{0}^{\infty }dr\;\rho_{\infty }\left(r\right)P_{\infty }(r+|t^{\prime }|)-A_{\infty }\int_{0}^{\infty }dr\;P_{\infty }\left(r\right)P_{\infty }(r+|t^{\prime }|), \end{array}$$

hence, finally

$$\left\langle \Sigma (t)\Sigma (t+s)\right\rangle =A_{\infty }\delta \left(s\right)-A_{\infty }\int_{0}^{\infty }dr\;P_{\infty }\left(r\right)P_{\infty }\left(r+|s|\right).$$

### Numerical implementation

The SPDE of Eq 7 can be readily integrated using the following numerical scheme:

$$\begin{array}{ll} q\left(t+\Delta t,r+\Delta t\right) & =q\left(t,r\right)\text{exp}\left\{-\rho \left[u\left(t,r\right)\right]\Delta t\right\}\\ & -\sqrt{\frac{q\left(t,r\right)H\left[q\left(t,r\right)\right]\left\{1-\text{exp}\left(-\rho \left[u\left(t,r\right)\right]\Delta t\right)\right\}}{N\Delta t}}N\left(0,1\right) \end{array}$$ (55)

where *H*(*x*) is the Heaviside step function, and N(0,1) is a standard normal random variable. This numerical scheme is obtained by discretizing the time evolution of *q*(*t*, *r*) on the characteristic curve (*cf.* Methods) and noting that

$$\begin{array}{c} 1-\rho qdt\approx e^{-\rho qdt}, \end{array}$$

with *e*^−*ρdt*^ the probability that a spike *is not* fired during interval *dt* [33]. The Heaviside function prevents negative values for *q*(*t*, *r*) from appearing under the square-root sign. Along the characteristic curve, the dynamics correspond to a Cox-Ingersoll-Ross stochastic differential equation [47]. The proposed numerical scheme above is thus well defined [48] and produces results in excellent agreement with simulations of the full network (see Fig 4). One way to extract the population activity is to evolve *q*(*t*, *r*) according to the above numerical scheme for all

$$\begin{array}{c} r=i\Delta t,\;\forall i>0, \end{array}$$

and compute *q*(*t*, 0)—and thus *A*(*t*)—by enforcing the conservation law (Eq 10).
